# Fibroblast Activation Protein Promotes Thoracic Aortic Dissection via PLAUR/ITGB1‐Mediated Pro‐inflammatory Macrophage Polarization

**DOI:** 10.1002/advs.202514786

**Published:** 2026-02-12

**Authors:** Hongqiao Zhu, Jianlie Wu, Ziyi Xu, Yifei Pei, Zaiping Jing, Jian Zhou, Rui Feng, Junjun Liu

**Affiliations:** ^1^ Department of Vascular Surgery Shanghai General Hospital Shanghai Jiao Tong University School of Medicine Shanghai China; ^2^ Department of Nursing Shanghai General Hospital Shanghai Jiao Tong University School of Medicine Shanghai China; ^3^ Department of Vascular Surgery Shanghai Fourth People's Hospital School of Medicine Tongji University Shanghai China; ^4^ Department of Vascular Surgery The First Affiliated Hospital Naval Medical University Shanghai China

**Keywords:** crosstalk, fibroblast activation protein, fibroblasts, macrophages, thoracic aortic dissection

## Abstract

**Background**: Thoracic aortic dissection (TAD) is a lethal vascular emergency lacking targeted therapies. Fibroblast activation protein (FAP), a protease implicated in tissue remodeling, exhibits unknown roles in TAD pathogenesis.

**Methods**: Human TAD specimens and β‐aminopropionitrile‐induced TAD models were used to assess FAP expression. Global and fibroblast‐specific Fap knockout mice were generated to evaluate biological effects of FAP. Bulk and single‐cell RNA sequencing were used to map cellular crosstalk and dysregulated signaling pathways. A combination of pharmacological inhibition (Ac‐Gly‐BoroPro), surface plasmon resonance (SPR), co‐immunoprecipitation (co‐IP), and functional rescue experiments was utilized to dissect the enzymatic versus nonenzymatic functions of FAP and its interaction with PLAUR/ITGB1 signaling.

**Results**: Fibroblast‐derived FAP was significantly upregulated in TAD lesions. Fap deletion markedly attenuated inflammatory infiltration, extracellular matrix degradation, and TAD incidence. Surprisingly, pharmacological inhibition of FAP's enzymatic activity failed to protect against TAD. SPR, co‐IP, and functional assays revealed that FAP binds directly to macrophage PLAUR via nonenzymatic sites. This interaction triggers ITGB1/FAK signaling, promoting a pro‐inflammatory macrophage phenotype that drives TAD progression.

**Conclusion**: This study demonstrates that FAP promotes TAD through a nonenzymatic mechanism involving fibroblast‐macrophage crosstalk via the FAP/PLAUR/ITGB1/FAK axis. Targeting this pathway might offer a promising therapeutic strategy for TAD.

## Graphical abstract

This study reveals that FAP promotes thoracic aortic dissection (TAD) through a nonenzymatic mechanism involving fibroblast‐macrophage crosstalk via the FAP/PLAUR/ITGB1/FAK axis. Targeting this pathway might offer a promising therapeutic strategy for TAD.

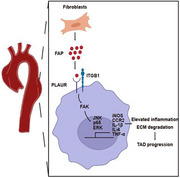

## 1 Introduction

Thoracic aortic dissection (TAD) is a life‐threatening aortic emergency characterized by high acute‐phase mortality and morbidity rates [1]. Currently, the medical management of TAD is confined to blood pressure control as the principal preventive and therapeutic approach, with a notable absence of targeted therapies [2]. This therapeutic gap highlights the critical need to elucidate the underlying pathogenic mechanisms. The pathogenesis of TAD involves structural failure of the aortic media, characterized by progressive degradation of the extracellular matrix (ECM). However, the precise molecular drivers of ECM degradation are still incompletely understood, presenting a major obstacle to translational therapeutic intervention.

Accumulating evidence indicates that fibroblast activation protein (FAP), a serine protease belonging to the dipeptidyl peptidase (DPP) family, is implicated in pathological tissue remodeling across diverse conditions [3]. Studies have shown that FAP is highly induced during inflammation and strongly expressed by mesenchymal cells of remodeling tissue [4, 5]. Brokopp et al. [6] demonstrated that the expression of FAP in human fibroatheromatous plaques is increased compared with plaque‐free aorta, and its expression is positively correlated with the stage of plaque progression. In recent years, FAP has been found to be highly expressed in abdominal aortic aneurysms (AAA) [7]. FAP's unique enzymatic activity and selective overexpression in pathological tissues render it a compelling candidate for targeted therapy. However, the expression profile, functional role, and underlying molecular mechanisms of FAP in TAD remain to be elucidated.

Our study aimed to delineate the stage‐specific role of FAP in TAD progression by integrating temporal profiling in a β‐aminopropionitrile (BAPN)‐induced mouse model with FAP genetic knockout and pharmacological inhibition. Combining bulk and single‐cell RNA sequencing, fibroblast‐macrophage co‐culture system, and in vivo functional validation, we elucidated the mechanism by which FAP regulates ECM degradation in TAD.

## 2 Materials and methods

The detailed description of materials and methods is provided in the Supporting Information.

### 2.1 Human Aortic Samples and Ethics Statement

Aortas from healthy donors and patients with TAD were collected from Shanghai General Hospital. Written informed consent was obtained from all patients for the use of their aortic tissue samples. Acute TAD was defined as occurring within 2 weeks of symptom onset, and chronic TAD was defined as occurring more than 2 months after onset. During surgery, full‐thickness segments of ascending aorta, including both dissected and nondissected areas, were immediately harvested. The protocol was approved by local Ethics Committee of Shanghai General Hospital (2025SQ131).

### 2.2 Animals

Animal studies were conducted under specific pathogen‐free barrier conditions and adhered to institutional guidelines. The study protocol was approved by the Ethics Committees of Shanghai General Hospital (2025AWS164). Additional details regarding the animals are available in the Supporting Information.

### 2.3 Statistical Analysis

Data are shown as mean ± standard deviation. Statistical comparisons between two groups were conducted after assessing normality using the Shapiro–Wilk test. For data adhering to a normal distribution, differences were evaluated with Student's *t*‐test; when an *F* test indicated unequal variances, Welch's correction was applied. Comparisons across three or more groups were performed using one‐way analysis of variance (ANOVA) followed by Tukey's post hoc test. For experiments involving two independent factors, a two‐way ANOVA was employed, with Bonferroni's post hoc analysis for detailed comparisons. Survival analysis was performed using Cox proportional‐hazards regression models. For all statistical analyses, two‐tailed tests were used, and a *p*‐value < 0.05 was considered statistically significant. The statistical analysis was performed using GraphPad Prism 9.0 and R 4.1.0.

## 3 Results

### 3.1 FAP Is Upregulated at the Site of TAD Lesions

**FIGURE 1:**
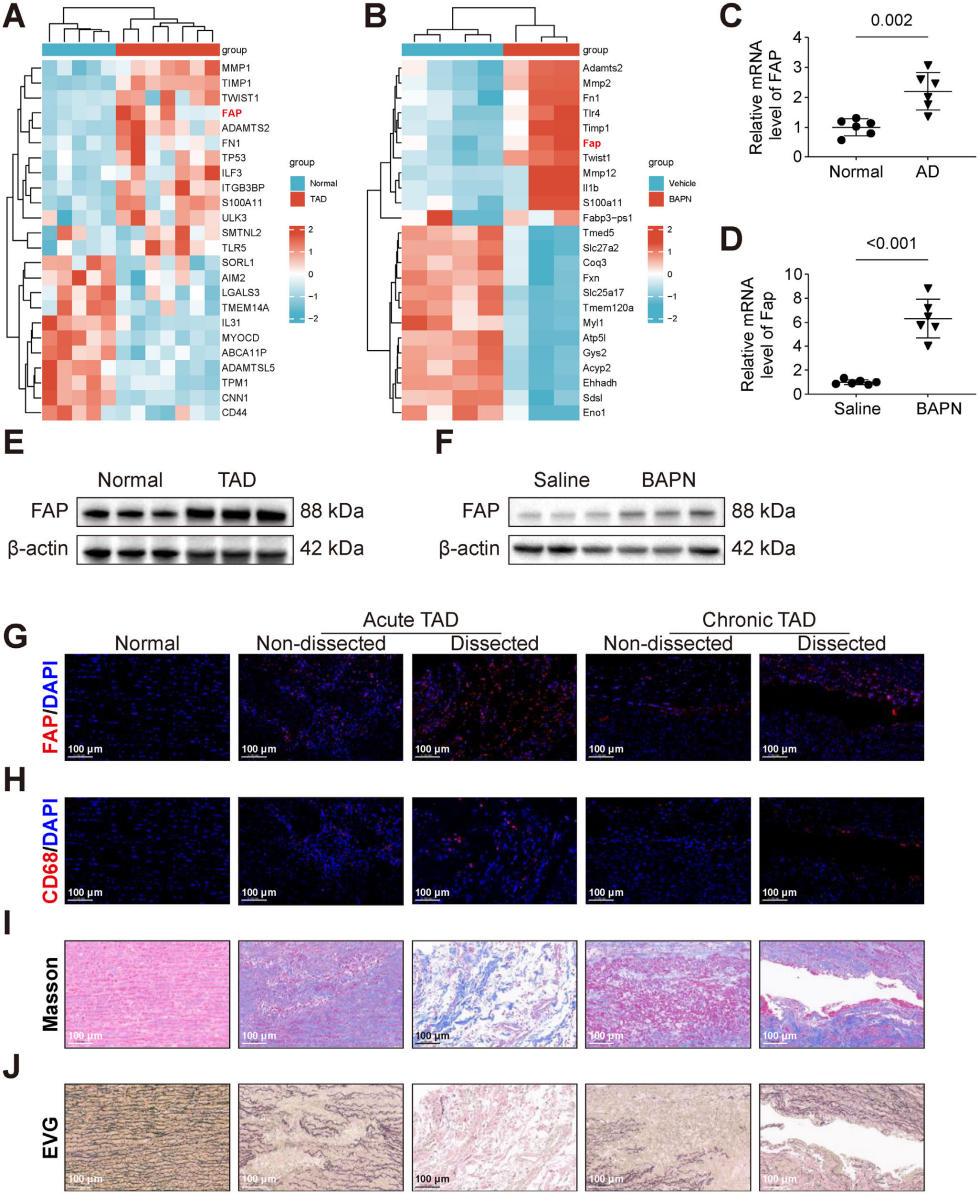
Fibroblast activation protein (FAP) is upregulated in thoracic aortic dissection (TAD) lesions and correlates with inflammatory infiltration and extracellular matrix (ECM) degradation. A) Heatmap showing differential gene expression profiles in human aortic tissues from healthy donors versus TAD patients (GSE52093; *p* < 0.05, |Log2FC| > 1). B) Heatmap showing distinct gene expression patterns in aortic specimens from control mice versus BAPN‐challenged mice (GSE247088; *p* < 0.05, |Log2FC| > 1). C) Relative FAP mRNA levels in human aortic tissues from healthy donors and TAD patients (*n* = 6 per group; unpaired two‐tailed *t*‐test). D) Relative Fap mRNA expression levels in aortic tissues from mice treated with saline or β‐aminopropionitrile (BAPN) for 28 days (*n* = 6 per group; Welch's *t*‐test). E) Relative FAP protein levels in human aortic tissues from healthy donors and TAD patients, as determined by Western blot (WB, *n* = 9 per group). F) Relative FAP protein levels in aortic tissues from saline‐ and BAPN‐treated mice, as determined by WB (*n* = 9 per group). G) Immunofluorescence staining for FAP (red) in human aortic tissues: normal aorta (from healthy donors), acute TAD lesion, adjacent nondissected region in acute TAD, chronic TAD lesion, and adjacent nondissected region in chronic TAD. Nuclei were counterstained with DAPI (blue). Scale bars, 100 µm. H) Immunofluorescence staining for CD68^+^ macrophages (red) in the corresponding regions described in (G). Nuclei were counterstained with DAPI (blue). Scale bars, 100 µm. I) Masson's trichrome staining of human aortic sections from the regions indicated in (G). Scale bars, 100 µm. J) Elastic van Gieson (EVG) staining of human aortic section from the regions indicated in (G). Scale bars, 100 µm.

Microarray and RNA‐seq expression profiles of aortic samples from TAD patients (GSE52093) and mouse TAD models (GSE247088) were retrieved from the Gene Expression Omnibus (GEO) database. Differential gene analysis was performed using R package with heatmap visualization. GEO datasets revealed upregulation of FAP in both TAD patients (Figure 1A) and mouse TAD models (Figure 1B). In aortic tissues from patients with TAD, the expression of FAP was significantly elevated compared with that in tissues from healthy donors, as demonstrated by quantitative polymerase chain reaction (qPCR) (Figure 1C) and Western blot (WB) analysis (Figures 1E and S1A). The expression levels of FAP were also significantly increased in the aortas of BAPN‐challenged mice, as evidenced by qPCR (Figure 1D) and WB analysis (Figures 1F and S1B). To investigate the spatial distribution of FAP expression in TAD, we performed immunofluorescence staining. Results revealed that within acute TAD, the fluorescent intensity of FAP was significantly higher in the dissected areas compared with the nondissected areas (Figures 1G and S1C). Similarly, in chronic‐phase dissections, regions of newly formed dissection exhibited increased FAP expression relative to the adjacent nondissected regions (Figures 1G and S1C). Locations with high FAP expression were also characterized by macrophage infiltration and ECM degradation, as evidenced by CD68 immunofluorescence (Figures 1H and S1D), Masson's trichrome (Figures 1I and S1E), and Elastic van Gieson (EVG) staining (Figures 1J and S1F). The aforementioned findings indicate that FAP expression is significantly upregulated in TAD lesions exhibiting inflammatory infiltration and ECM degradation, suggesting its potential involvement in the underlying pathogenic mechanisms.

### 3.2 Fibroblast‐Derived FAP Is Upregulated at Early Stages of TAD

**FIGURE 2:**
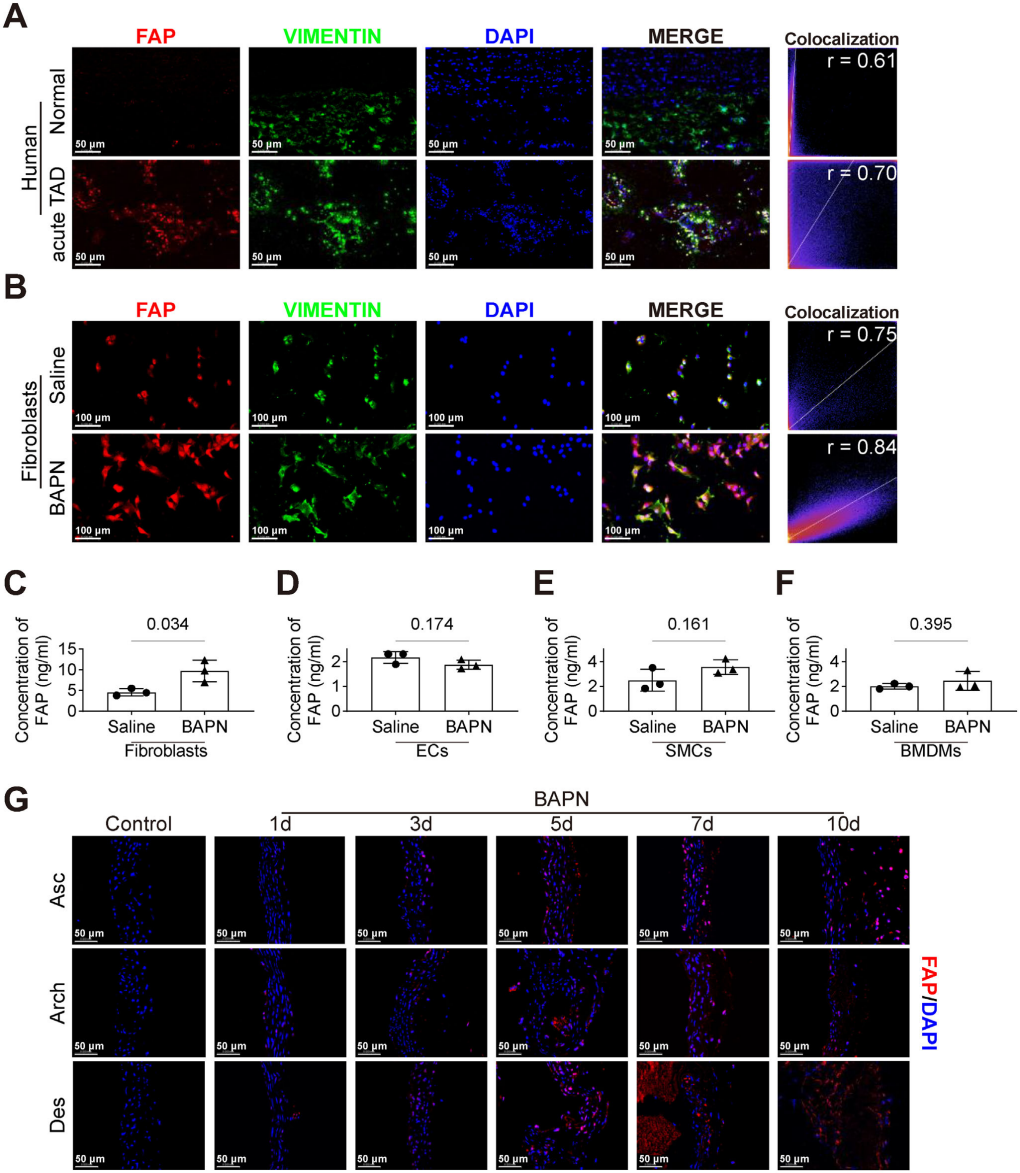
Fibroblast‐derived FAP is upregulated at early stages of BAPN‐induced TAD model. A) Representative immunofluorescence images of human aortic tissues from a normal donor and a patient with acute TAD. Tissues were stained for FAP (red), VIMENTIN (green), and nuclei (DAPI, blue). Quantification of colocalization using Pearson's correlation coefficient (*r*). Scale bars, 50 µm. B) Representative immunofluorescence images of aortic fibroblasts from mice administered with saline or BAPN for 28 days. Cells were stained for FAP (red), VIMENTIN (green), and nuclei (DAPI, blue). Quantification of colocalization using Pearson's correlation coefficient (*r*). Scale bars, 100 µm. C–F) Quantification of FAP protein levels in supernatants derived from fibroblasts (C), endothelial cells (ECs, D), smooth muscle cells (SMCs, E), and bone marrow‐derived macrophages (BMDMs, F), each isolated from mice treated with either saline or BAPN for 28 days and cultured for 24 h (*n* = 3 per group, unpaired two‐tailed *t*‐test). G) Time‐course immunofluorescence showing FAP (red) in the aortic tissues (ascending aorta, aortic arch, and descending aorta) from mice at 1, 3, 5, 7, and 10 days post‐BAPN administration and saline control. Nuclei are counterstained with DAPI (blue). Scale bars, 50 µm.

Utilizing single‐cell RNA sequencing data of aortic samples from TAD patients (GSE213740) and mouse TAD models (PRJCA003113), we investigated the cellular origin of FAP. Results revealed that FAP expression was specifically enriched in fibroblasts and that the proportion of FAP^+^ fibroblasts was significantly increased in TAD aortic samples (Figure S2A–F). Immunofluorescence staining of human aortic tissues demonstrated strong colocalization of the fibroblast marker VIMENTIN with FAP (Figure 2A), whereas no strong co‐localization was observed between FAP and CD31 (endothelial cells, ECs), αSMA (smooth muscle cells, SMCs), or CD68 (macrophages) (Figure S3A–C). To further validate fibroblasts as the primary source of FAP in TAD, a BAPN‐induced mouse TAD model was established, and ECs, fibroblasts, SMCs, and bone marrow‐derived macrophages (BMDMs) were isolated from the aorta and femur for immunofluorescence staining and enzyme‐linked immunosorbent assay (ELISA) (Figure S4A). Immunofluorescence staining confirmed strong co‐localization of FAP in mouse aortic fibroblasts (Figure 2B), but not with ECs, smooth muscle cells, or BMDMs (Figure S4B–D). FAP levels were significantly elevated in fibroblasts’ supernatant from the BAPN group compared with the Saline group (Figure 2C), while no such increase was observed in ECs, SMCs, or BMDMs (Figure 2D–F). These results indicate that fibroblasts are the predominant source of FAP and that its secretion is markedly upregulated in TAD.

To characterize the temporal dynamics of fibroblast‐derived FAP during TAD progression, we first analyzed public single‐cell RNA sequencing datasets. Interrogation of a human aortic single‐cell dataset (GSE222318) revealed that FAP expression in fibroblasts was upregulated from the acute to the subacute phase and downregulated in the chronic phase (Figure S2G). In a BAPN‐induced mouse TAD model (PRJCA003113), Fap expression in fibroblasts was elevated at day 7 post‐induction and remained high at day 14 and 21 (Figure S2H). To further examine whether fibroblast‐derived FAP is upregulated early in BAPN‐induced TAD, we conducted a time‐course experiment in which mice were subjected to BAPN and tissues were harvested at day 1, 3, 5, 7, and 10. Segmental analysis revealed that FAP^+^ signals were first detected on one‐day BAPN administration in the ascending aorta and aortic arch, and on day 3 in the descending aorta, with a progressive increase thereafter in all segments (Figures 2G and S5A–C). F4/80^+^ signals were significantly elevated in the ascending and descending aorta on day 7, and in the aortic arch on day 5 following BAPN administration (Figure S5D–G). The relative collagen volume fraction and elastin degradation score, as determined by Masson's trichrome and EVG staining, were significantly increased on the early time point of BAPN induction (Figures S6A–D and S7A–D). Collectively, these findings suggest that fibroblast‐derived FAP is upregulated during early stages of TAD and is associated with inflammatory infiltration and ECM degradation.

### 3.3 Fap Deletion Mitigates Early Inflammatory Infiltration and ECM Degradation in BAPN‐Induced TAD

**FIGURE 3:**
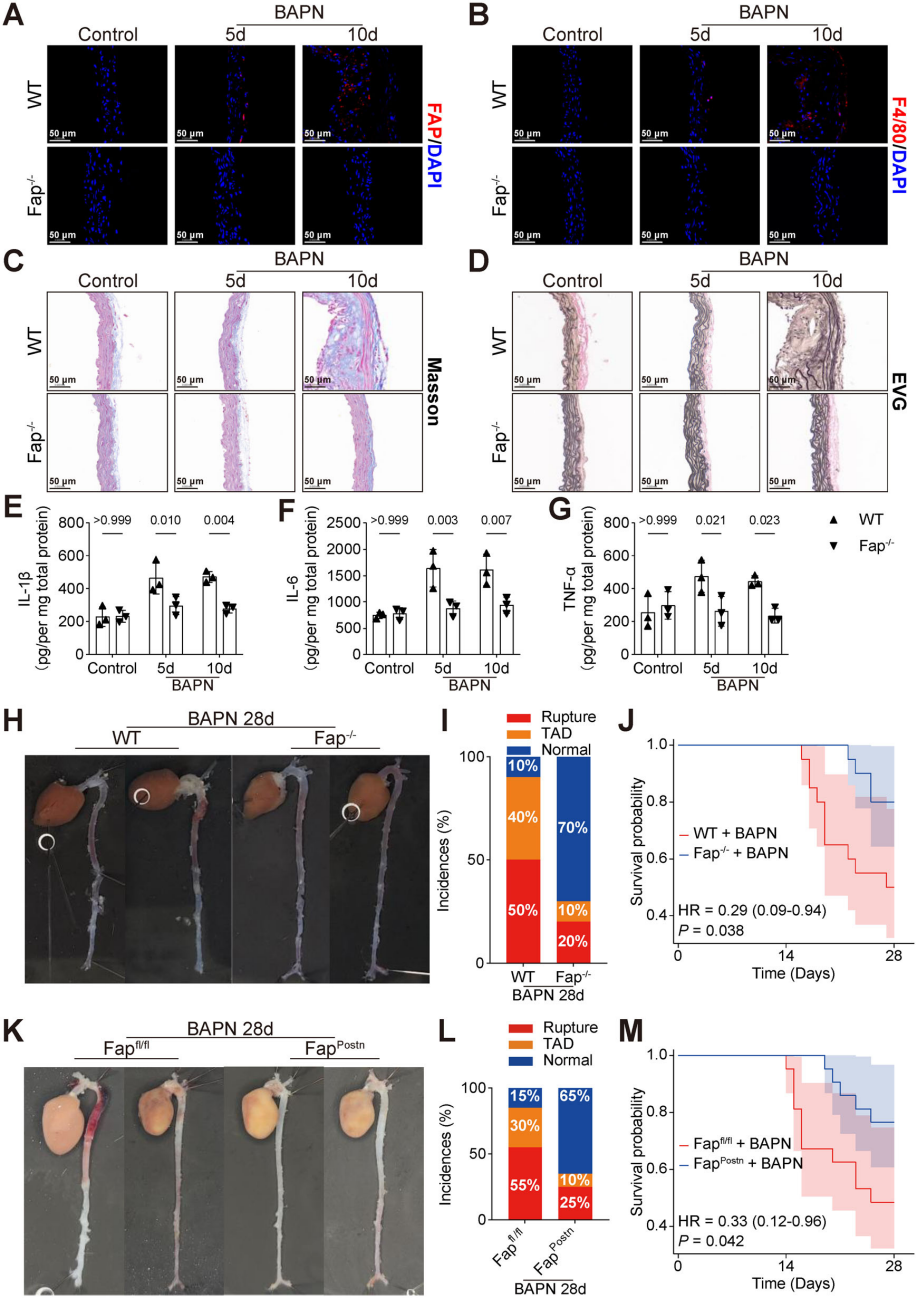
Fap deletion attenuates early inflammatory infiltration and ECM degradation to prevent TAD. A, B) Representative immunofluorescence staining for FAP (A) and F4/80 (B) (red) in aortic sections from wild‐type (WT) and Fap^−/−^ mice treated with saline (control) or BAPN for 5 or 10 days. Nuclei are counterstained with DAPI (blue). Scale bars, 50 µm. C,D) Representative histological staining of aortic sections using Masson's trichrome (C) and EVG (D) from WT and Fap^−/−^ mice at indicated time points. Scale bars, 50 µm. E–G) Interleukin (IL)‐1β (E), IL‐6 (F), and tumor necrosis factor‐alpha (TNF‐α) (G) in aortic samples of WT and Fap^−/−^ mice were quantified by enzyme‐linked immunosorbent assay (ELISA). Mice were treated with saline for 10 days (Control) or with BAPN for 5 or 10 days (*n* = 3 per group each time point; two‐way ANOVA with Bonferroni multiple comparison test). H) Representative macroscopic images of aortas from WT and Fap^−/−^ mice following 28‐day BAPN administration. I) Stacked bar chart showing the incidence of normal aorta (blue), TAD (orange), and rupture (red) in the WT and Fap^−/−^ mice following 28‐day BAPN administration (*n* = 20 per group). J) Survival probability between the WT and Fap^−/−^ groups was compared by Cox proportional hazards model (*n* = 20 per group). K) Representative macroscopic images of aortas from Fap^fl/fl^; and Fap^fl/fl^;Postn‐Cre (Fap^Postn^) mice following 28‐day BAPN administration. L) Stacked bar chart showing the incidence of normal aorta (blue), TAD (orange), and rupture (red) in the Fap^fl/fl^ and Fap^Postn^ groups (*n* = 20 per group). M) Survival probability between the Fap^fl/fl^ and Fap^Postn^ groups was compared by Cox proportional hazards model (*n* = 20 per group).

To investigate whether FAP plays a critical role in ECM degradation and inflammatory responses during early stages of BAPN‐induced TAD, we utilized global knockout (Fap^−/−^) mice (Figure S8A,B). Wild‐type (WT) and Fap^−/−^ mice were challenged with BAPN and aortic tissues were collected for analyzed on days 5 and 10. Immunofluorescence staining revealed significantly reduced fluorescent intensity of FAP and F4/80 in the aortas of Fap^−/−^ mice compared with WT controls at both time points (Figures 3A,B and S8C,D). Histological analysis, including EVG and Masson staining, demonstrated that collagen deposition and elastin breaks were markedly ameliorated in Fap^−/−^ mice, compared with the WT group (Figures 3C,D and S8E,F). Further quantification of inflammatory cytokines via ELISA at day 5 and 10 showed consistently lower levels of Interleukin (IL)‐6, IL‐1β, and tumor necrosis factor‐alpha (TNF‐α) in Fap^−/−^ mice, compared with WT mice (Figure 3E–G). These findings suggest that Fap deletion alleviates early inflammatory responses and ECM degradation in TAD pathogenesis. To evaluate whether Fap ablation confers protection against TAD progression, we extended the observation period to 28 days with a larger cohort (*n* = 20 per group). Results indicated that Fap deletion significantly reduced the incidence of TAD, aortic rupture, and mortality (Figures 3H–J and S9A–J). To delineate the contribution of fibroblast‐specific FAP, we generated fibroblast‐specific conditional knockout mice (Fap^fl/fl^; Postn^cre/cre^, termed Fap^Postn^) and subjected them to 28‐day BAPN intervention (Figure S10A,B). Consistent with the global knockout phenotype, fibroblast‐specific Fap deletion also conferred protection against TAD (Figure 3K–M and S10C–L). Collectively, these data demonstrate that Fap deficiency mitigates early inflammatory infiltration and ECM degradation, thereby preventing the development of TAD. However, the underlying mechanisms require further investigation.

### 3.4 Pharmacological Inhibition of FAP Enzymatic Activity Is Insufficient to Prevent BAPN‐Induced TAD

The structural integrity of the ECM plays a pivotal role in preserving aortic wall homeostasis [8]. It was observed that FAP demonstrates weak DPP activity [10]. Notably, DPP4, a related enzyme with strong DPP activity, has been associated with AAA progression [11]. In addition, FAP exhibits endopeptidase activity against substrates such as denatured type I collagen and gelatin, suggesting its potential involvement in ECM remodeling during TAD pathogenesis [9]. The boronic acid‐based inhibitor Ac‐Gly‐BoroPro, structurally derived from the N‐acyl‐Gly‐Pro motif, demonstrates high specificity in inhibiting both the DPP and endopeptidase activities of FAP [12, 13]. Given the critical role of ECM degradation in TAD, the role of FAP enzymatic activity in TAD pathogenesis warrants further investigation.

To evaluate the inhibitory efficacy of FAP enzymatic activity, WT mice were administered Ac‐Gly‐BoroPro at doses of 50 or 500 µg/kg/day. Aortic samples were collected at multiple time points (day 7, 14, 21, and 28) following BAPN induction. FAP protein levels in aortic tissues from each experimental group were quantified using ELISA. Enzymatic activity was evaluated with an FAP‐specific fluorescence‐based kit. The results demonstrated that treatment with Ac‐Gly‐BoroPro did not significantly alter the BAPN‐induced increase in FAP protein levels (Figure S11A). In contrast, treatment with 500 µg/kg/day Ac‐Gly‐BoroPro markedly suppressed the BAPN‐induced upregulation of enzymatic activity of FAP, reducing it to a level comparable to that in the control group (Figure S11B). These results indicate that Ac‐Gly‐BoroPro effectively inhibits the proteolytic activity of FAP without affecting its protein expression levels.

**FIGURE 4:**
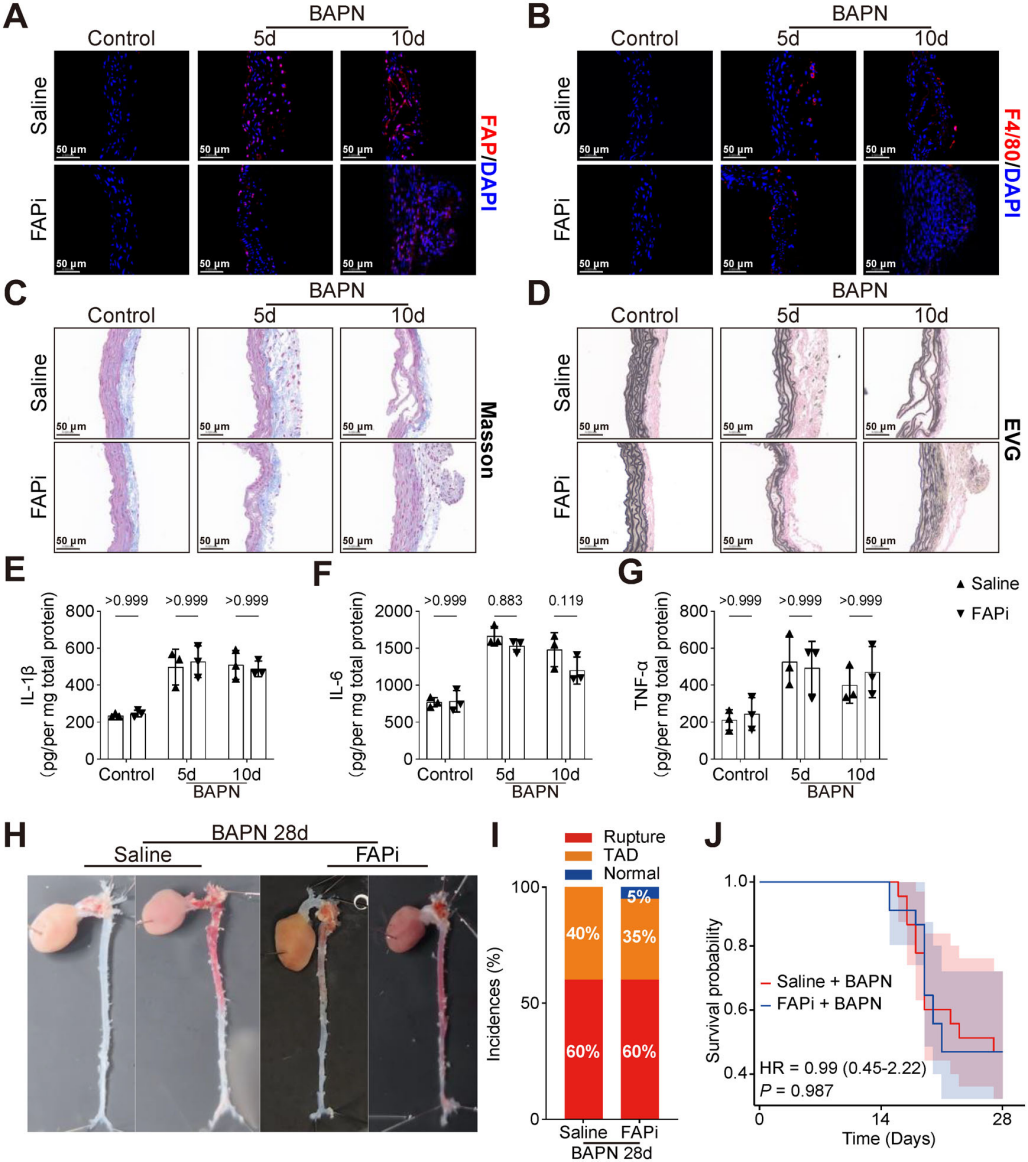
FAP inhibition fails to protect mice against BAPN‐induced TAD formation. A,B) Representative immunofluorescence images of FAP (A, red) and F4/80 (B, red) in aortic sections from Saline group (3‐week WT male mice treated with saline) and FAPi group (3‐week WT male mice treated with 500 µg/kg/day Ac‐Gly‐BoroPro) under control conditions or after 5 or 10 days of BAPN administration. Nuclei are counterstained with DAPI (blue). Scale bars, 50 µm. C,D) Representative histological staining (Masson [C], EVG [D]) of aortic sections in each group. Scale bars, 50 µm. E‐G) IL‐1β (E), IL‐6 (F), and TNF‐α (G) in aortic samples of the Saline and FAPi groups were quantified by ELISA. Mice were treated with saline for 10 days (Control) or with BAPN for 5 or 10 days (*n* = 3 per group each time point; two‐way ANOVA with Bonferroni multiple comparison test). H) Representative macroscopic images of aortas from the Saline and FAPi groups following 28‐day BAPN administration. I) Stacked bar chart showing the incidence of normal aorta (blue), TAD (orange), and rupture (red) in the Saline and FAPi groups (*n* = 20 per group). J) Survival probability between the Saline and FAPi groups was compared by Cox proportional hazards model (*n* = 20 per group).

To determine whether Ac‐Gly‐BoroPro intervention alleviates early inflammatory responses and ECM degradation induced by BAPN, 3‐week WT male mice were divided into two groups: a Saline group (*n* = 12) and a treatment group receiving 500 µg/kg/day Ac‐Gly‐BoroPro (FAPi group, *n* = 12). Both groups underwent BAPN induction, and tissues were collected on day 5 (*n* = 6 per group) and 10 (*n* = 6 per group) for analysis via immunofluorescence staining, histological examination, and ELISA. The results indicated that, compared with the Saline group, treatment with Ac‐Gly‐BoroPro did not significantly reduce BAPN‐induced aortic inflammation or ECM degradation at either time point (Figures 4A–G and S12A–D). To further evaluate whether Ac‐Gly‐BoroPro confers protection against the development of TAD, 3‐week WT male mice (Saline and FAPi group, *n* = 20 per group) were subjected to BAPN induction for 28 days. Results revealed that Ac‐Gly‐BoroPro treatment failed to prevent the formation of TAD (Figures 4H–J and S13A–J). These results suggest that FAP might modulate ECM remodeling through mechanisms independent of its enzymatic activity, underscoring the need for comprehensive investigation into its target‐specific regulatory networks.

### 3.5 FAP^+^ Fibroblasts Interact With IL1B^+^ Macrophages Through Ligand–Receptor Interactions Involving PLAUR and ITGB1

**FIGURE 5:**
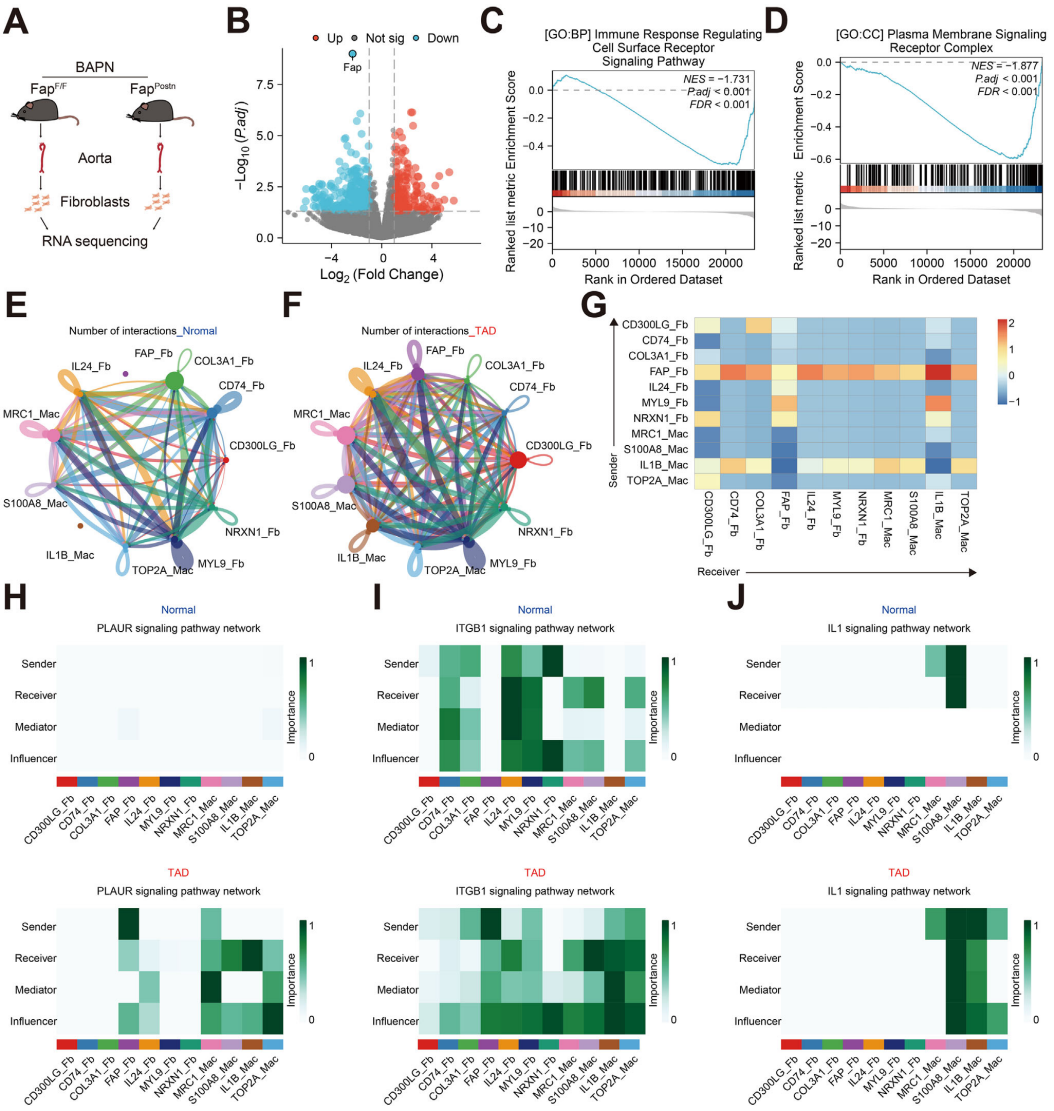
FAP^+^ fibroblasts communicates with IL1B^+^ macrophages through PLAUR and ITGB1 signaling pathways. A) Fibroblasts from the aortas of BAPN‐treated Fap^fl/fl^ and Fap^postn^ mice were isolated for RNA sequencing (*n* = 3 per group). B) Volcano plot displaying 197 upregulated and 360 downregulated differentially expressed genes (|Log2FC| > 1, adj. *p* < 0.05) after Fap deletion. C) Enrichment analysis of Gene Ontology: Biological Process (GO: BP) revealed that Fap knockout significantly downregulated the immune response‐regulating cell surface receptor signaling pathway in fibroblasts. D) GO: Cellular Component (GO: CC) enrichment analysis demonstrated that Fap knockout significantly suppressed plasma membrane signaling receptor complexes in fibroblasts. E,F) The signaling networks illustrate seven fibroblast subsets and four macrophage subsets in Normal and TAD groups, with circle sizes corresponding to cell population counts and edge widths reflecting interaction probabilities (GSE213740). G) Heatmap displaying pathway alterations in fibroblast‐macrophage interactions between Normal and TAD groups (GSE213740). H–J) Heatmap illustrating the relative contributions of fibroblasts and macrophages to PLAUR, ITGB1, and IL1 signaling pathways in Normal versus TAD groups. PLAUR: plasminogen activator urokinase receptor; ITGB1: integrin beta‐1.

To further investigate the regulatory mechanisms of FAP^+^ fibroblasts in TAD, aortic samples from Fap^fl/fl^ and Fap^Postn^ mice challenged by BAPN were harvested, and fibroblasts were subsequently isolated for bulk RNA sequencing (Figure 5A). Transcriptomic profiling identified 197 upregulated and 360 downregulated differentially expressed genes (|logFC| >1, adj. *p* < 0.05) following Fap ablation (Figure 5B). Gene set enrichment analysis of Gene Ontology (GO) identified significant downregulation of the immune response‐regulating cell surface receptor signaling pathway and plasma membrane signaling receptor complex in Fap^Postn^ mice's fibroblasts (Figure 5C,D). These findings suggest that FAP^+^ fibroblasts might influence TAD progression via modulation of immune responses. To further investigate how FAP^+^ fibroblasts regulate immune responses through extracellular signaling pathways, we performed CellChat analysis on publicly available single‐cell sequencing data of human TAD (GSE213740). Our analysis revealed that, compared with the Normal samples, there was a significantly enhanced interaction propensity between FAP^+^ fibroblasts and macrophages (Figures S2E,F and S14A) in the TAD samples (Figure 5E,F). Our analysis identified FAP^+^ fibroblasts primarily functioning as signaling sources for macrophage subtypes, with the highest interaction probability observed in their communication with IL1B^+^ macrophages (Figure 5G). Subsequent analyses established that the IL1B^+^ macrophages exhibit a pro‐inflammatory phenotype, marked by predominant expression of IL1B, HIF1A, ENO1, and related genes (Figure S14A). Compared with Normal samples, TAD samples’ PLAUR (plasminogen activator urokinase receptor) and ITGB1 (integrin beta‐1) signaling pathways were upregulated in communication networks where FAP^+^ fibroblasts functioned as sender cells and IL1B^+^ macrophages served as receiver cells (Figure 5H–J). Notably, IL1B^+^ macrophages exhibited specific high expression of both PLAUR and ITGB1 (Figure S14B), suggesting their potential role as key responders to fibroblast‐derived signals within these activated pathways. We further validated the co‐localization of FAP, PLAUR, and ITGB1 via immunofluorescence staining in human aortic tissues and BAPN‐induced mouse aortic lesions, confirming their spatial co‐localization (Figure S15A,B). Based on these results, we propose that FAP^+^ fibroblasts might promote TAD progression through specific ligand–receptor interactions with pro‐inflammatory IL1B^+^ macrophages. However, the exact binding mechanisms between fibroblast‐derived FAP and macrophage PLAUR, including binding sites and functional effects, remain to be elucidated.

### 3.6 FAP Drives Inflammation and ECM Degradation via Nonenzymatic Site‐Mediated Binding to Macrophage PLAUR

**FIGURE 6:**
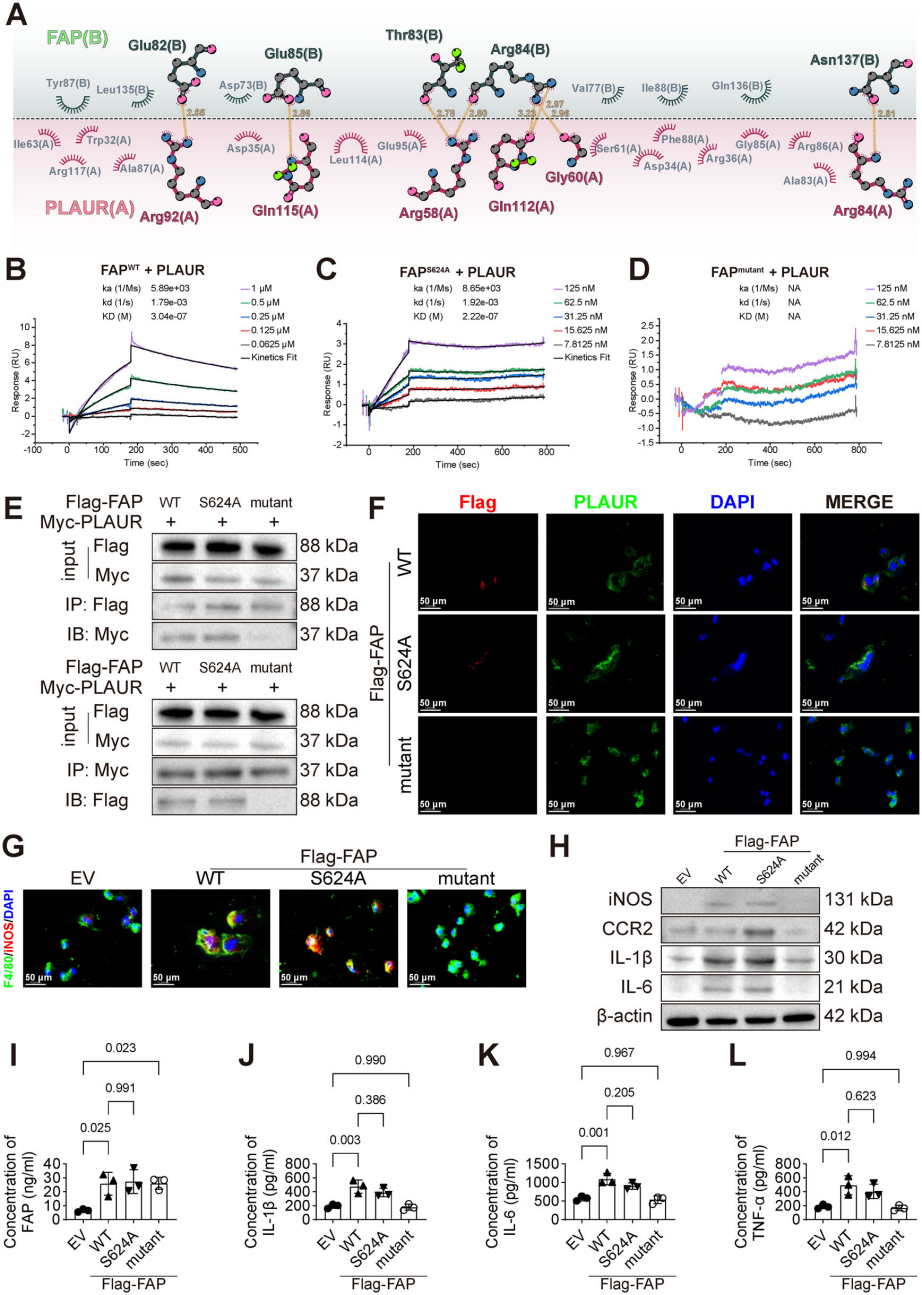
FAP binds to PLAUR via nonenzymatic sites and promotes macrophage pro‐inflammatory polarization. A) Molecular model of the FAP/PLAUR interface, with FAP depicted as chain B and PLAUR as chain A, showing key residues and intermolecular distances. B–D) The surface plasmon resonance analyses for binding kinetics between FAP^WT^ (B), FAP^S624A^ (C), and FAP^mutant^ (D) and PLAUR. E) Co‐immunoprecipitation (co‐IP) assay performed in 293T cells co‐transfected with Flag‐tagged FAP and FAP mutants (FAP^WT^, FAP^S624A^, and FAP^mutant^) and Myc‐tagged PLAUR to examine the interaction between FAP and PLAUR. IP was conducted with anti‐Flag antibody followed by immunoblotting (IB) with anti‐Myc antibody, and reciprocally, with anti‐Myc antibody for IP and anti‐Flag antibody for IB. F) Fibroblasts were transfected with Flag‐tagged proteins (FAP^WT^, FAP^S624A^, and FAP^mutant^) and co‐cultured with macrophages. Representative immunofluorescence images of Flag (red) and PLAUR (green) colocalization in macrophages. Nuclei were counterstained with DAPI (blue). Scale bars, 50 µm. G) Representative immunofluorescence images of F4/80 (green) and iNOS (red) in macrophages co‐cultured with fibroblasts, which were transfected with empty vector (EV), FAP^WT^, FAP^S624A^ and FAP^mutant^. Nuclei were counterstained with DAPI (blue). Scale bars, 50 µm. H) WB analysis of iNOS, CCR2, IL‐1β, and IL‐6 in macrophages co‐cultured with each transfected fibroblast group (*n* = 3 per group). I–L) ELISA of FAP, IL‐1β, IL‐6, and TNF‐α in supernatant from macrophages co‐cultured with each transfected fibroblast group (*n* = 3 per group; one‐way ANOVA with Tukey's post‐hoc test). ka: association rate constant; kd: dissociation rate constant; KD: dissociation constant.

Based on prior findings indicating a correlation between FAP and PLAUR, we conducted further investigations to delineate the specific binding sites between FAP and PLAUR and to examine how their interaction modulates the pro‐inflammatory phenotype of macrophages. Initially, molecular docking was employed to preliminarily assess the binding conformation and interacting residues of the FAP/PLAUR complex. Subsequently, we utilized Alphafold3 to reconstruct the FAP/PLAUR complex model and employed molecular dynamics (MD) simulations to analyze the dynamic stability of the complex (Figure S16A). Throughout the 100 ns MD simulation, the complex rapidly reached a stable plateau, with the average root‐mean‐square deviation (RMSD) stabilizing at approximately 3.13 Å (Figure S16B). We calculated the binding free energy of the complex using the Molecular Mechanics/Generalized Born Surface Area method. The computed total binding free energy (ΔG) was −37.90 kcal/mol, a strongly negative value indicative of a highly stable and high‐affinity interaction between PLAUR and FAP (Figure S16C). The two‐dimensional interaction analysis revealed that five key residues in FAP (Glu82, Glu85, Thr83, Arg84, and Asn137) engage with six residues in PLAUR (Arg92, Gln115, Arg58, Gln112, Gly60, and Arg84) via hydrogen bonding. These hydrogen bonds were measured at distances of 2.85, 2.86, 2.78, 2.80, 3.23, 2.97, 2.96, and 2.81 Å, respectively (Figure 6A). To experimentally validate the precise binding sites, we generated a multisite mutant FAP (Glu82Ala, Thr83Ala, Arg84Ala, Glu85Ala, Ser86Ala, and Tyr87Ala; termed FAP^mutant^) based on the computational predictions, specifically targeting the proposed interaction interface. In addition, we constructed an enzymatic site mutant of FAP (Ser624Ala, termed FAP^S624A^) to specifically assess the contribution of FAP's proteolytic activity to PLAUR binding. Surface plasmon resonance (SPR) assays demonstrated that the dissociation constant (KD) for FAP^WT^ binding to PLAUR was 3.04 × 10^−7 ^M (Figure 6B), while that for FAP^S624A^ binding to PLAUR was 2.22 × 10^−7^ M (Figure 6C). In contrast, the KD for FAP^mutant^ binding to PLAUR cannot be determined (Figure 6D). Furthermore, co‐immunoprecipitation (co‐IP) experiments in 293T cells expressing Flag‐tagged FAP mutants and Myc‐tagged PLAUR were conducted. WB analysis revealed that only Flag‐FAP^mutant^ showed no detectable interaction with PLAUR, indicating that the mutant sites are critical for the protein–protein interaction (Figure 6E). To further investigate the interaction between fibroblast‐derived FAP and macrophage PLAUR, we established a fibroblast‐macrophage co‐culture system. Aortic fibroblasts from Fap^−/−^ mice were transduced to overexpress Flag‐tagged FAP^WT^, FAP^S624A^, or FAP^mutant^, and then co‐cultured with macrophages. Immunofluorescence analysis confirmed that Flag‐FAP^mutant^, but not Flag‐FAP^WT^ or Flag‐FAP^S624A^, disrupted the FAP/PLAUR interaction (Figure 6F). These results demonstrate that FAP may bind to PLAUR specifically through nonenzymatic sites.

We next examined the functional consequence of the FAP/PLAUR interaction on macrophage pro‐inflammatory polarization. Fibroblasts overexpressing Flag‐FAP^WT^, FAP^S624A^, or FAP^mutant^ were co‐cultured with macrophages. The macrophages were subsequently analyzed using immunofluorescence staining, WB and ELISA. The results indicated that FAP^mutant^ lacks the ability to promote macrophage‐mediated inflammation, unlike FAP^WT^ or FAP^S624A^ (Figures 6G–L and S16D,E).

**FIGURE 7:**
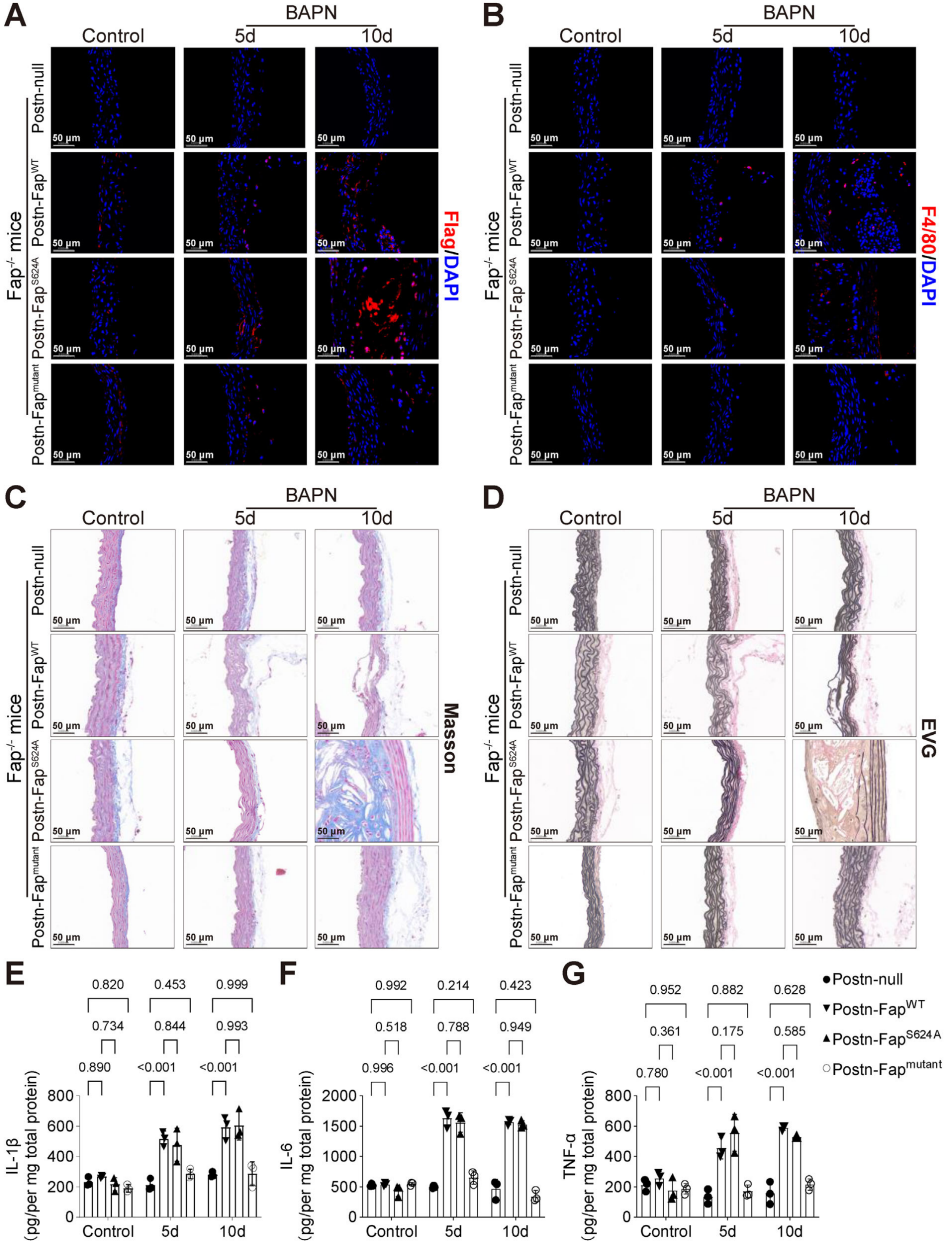
In vivo disruption of the FAP/PLAUR interaction attenuates BAPN‐induced early aortic inflammation and ECM degeneration A,B) Mice were injected with recombinant adeno‐associated virus serotype 9 (rAAV9) carrying Postn‐promoter‐driven FAP^WT^, FAP^S624A^, or FAP^mutant^ constructs with a C‐terminal Flag. Aortas of each group were harvested under control conditions or after BAPN administration for 5 or 10 days. Immunofluorescence images showing Flag (red, A) and F4/80 (red, B) staining in aortic tissues of each group at different time points. Nuclei were counterstained with DAPI (blue). Scale bars, 50 µm. C,D) Representative histological staining (Masson [C], EVG [D]) of aortic tissues in each group at different time points. Scale bars, 50 µm. E–G) ELISA of IL‐1β (E), IL‐6 (F), and TNF‐α (G) from aortic tissues in each group (*n* = 3 per group each time point; two‐way ANOVA with Bonferroni multiple comparison test).

To delineate the in vivo role of the nonenzymatic sites of fibroblast‐derived FAP in the pathogenesis of TAD, we specifically overexpressed FAP^WT^, FAP^S624A^, or FAP^mutant^ in aortic fibroblasts of Fap^−/−^ mice. This was achieved via injection of recombinant adeno‐associated virus serotype 9 (rAAV9) carrying Postn‐promoter‐driven FAP^WT^, FAP^S624A^, or FAP^mutant^ constructs with a C‐terminal Flag tag into 18‐day‐old Fap^−/−^ male mice; a null vector was used as control. After one week, mice were administered with BAPN for 5 or 10 days (Figure S17A). Immunofluorescence analysis revealed comparable Flag fluorescence intensity across the FAP^WT^, FAP^S624A^, and FAP^mutant^ groups in control, BAPN‐5‐day, and BAPN‐10‐day cohorts (Figures 7A and S17B). However, mice in the FAP^mutant^ group exhibited significantly reduced collagen deposition, elastin breaks, and inflammation after both 5 and 10 days of BAPN administration compared with the FAP^WT^ and FAP^S624A^ groups (Figures 7B–G and S17C–E). These results demonstrate that fibroblast‐derived FAP binding to PLAUR via nonenzymatic sites modulates macrophage pro‐inflammatory polarization, thereby promoting early inflammatory responses and ECM degradation.

### 3.7 Fibroblast‐Derived FAP Modulates Macrophage Pro‐inflammatory Phenotype via PLAUR/ITGB1/FAK Pathway

**FIGURE 8:**
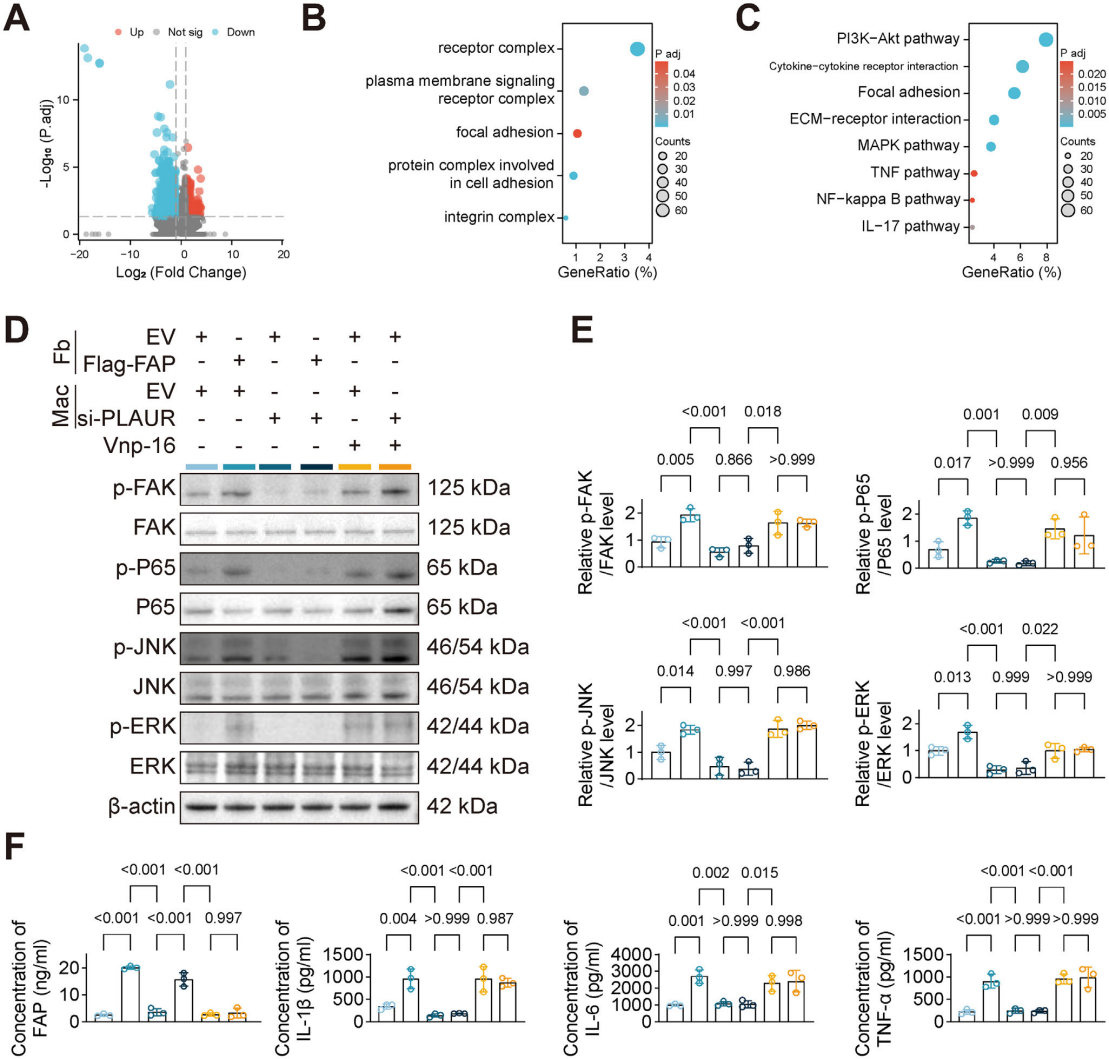
Fibroblast‐derived FAP modulates macrophage pro‐inflammatory phenotype via PLAUR/ITGB1/FAK pathway. A) RNA sequencing analysis revealed 1537 downregulated and 213 upregulated genes (|log2FC| > 1, adj. *p* < 0.05) in macrophages co‐cultured with Fap^ −/−^ fibroblasts transfected with FAP or EV (*n* = 3 per group). B) GO: BP enrichment analysis of downregulated genes demonstrated significant decrease in macrophages co‐cultured with fibroblasts transfected with EV, compared with the FAP‐overexpressed fibroblasts. C) Kyoto Encyclopedia of Genes and Genomes pathway analysis showed significant downregulation signaling pathways in macrophages co‐cultured with fibroblasts transfected with EV, compared with the FAP‐overexpressed fibroblasts. D,E) WB (D) of key signaling pathway proteins in macrophages co‐cultured with each fibroblast group. Quantification analysis (E) showed that FAP overexpression significantly increased phosphorylation of FAK, p65, JNK, and ERK in macrophages. This effect was abrogated by PLAUR knockdown but rescued by treatment with the ITGB1 agonist Vnp‐16 (*n* = 3 per group; one‐way ANOVA with Tukey's post hoc test). F) The protein levels of FAP, IL‐1β, IL‐6, and TNF‐α in macrophage culture supernatants from each group were quantified by ELISA (*n* = 3 per group; one‐way ANOVA with Tukey's post hoc test).

To further investigate the downstream signaling pathways through which fibroblast‐derived FAP regulates the pro‐inflammatory phenotype in macrophages, we established a fibroblast‐macrophage co‐culture system. Aortic fibroblasts isolated from Fap^ −/−^ mice were transduced to overexpress either FAP or a control empty vector (EV), and subsequently co‐cultured with macrophages. RNA sequencing analysis of these macrophages identified 1537 downregulated and 213 upregulated genes (|log2FC| >1, adj. *p* < 0.05) (Figure 8A). GO enrichment analyses of downregulated genes revealed that macrophages co‐cultured with Fap^−/−^ fibroblasts exhibited significant downregulation of pathways related to receptor complex, plasma membrane signaling receptor complex, focal adhesion, protein complex involved in cell adhesion, and integrin complex (Figure 8B). Kyoto Encyclopedia of Genes and Genomes pathway analysis demonstrated that Fap^−/−^ fibroblasts significantly downregulated the Focal adhesion, MAPK, NF‐κB, and TNF signaling pathways in macrophages (Figure 8C). Previous studies have demonstrated that integrins could activate the FAK (Focal adhesion kinase)‐NF‐κB signaling cascade, thereby amplifying pro‐inflammatory cytokine production during innate immune responses [14]. Evidence from cancer research suggests that FAP may activate the integrin/FAK signaling pathway through its interaction with PLAUR [10]. However, the precise mechanism by which FAP regulates macrophage pro‐inflammatory phenotype through this signaling pathway remains unclear.

Previous studies have shown that FAP binding to PLAUR facilitates the subsequent interaction between PLAUR and ITGB1 [15]. We investigated whether FAP activates ITGB1 through PLAUR, leading to downstream FAK signaling activation. Using the fibroblast‐macrophage co‐culture system, we overexpressed FAP or an EV in fibroblasts and knocked down PLAUR in macrophages using siRNA (Figure S18). WB analysis of macrophage lysates showed that FAP overexpression enhanced phosphorylation of FAK, p65, JNK, and ERK. However, PLAUR knockdown abrogated this activation. Notably, treatment with the ITGB1 agonist Vnp‐16 rescued the suppression of FAK, p65, JNK, and ERK phosphorylation caused by PLAUR knockdown (Figure 8D,E). ELISA of macrophage culture supernatants revealed that FAP overexpression significantly increased the secretion of pro‐inflammatory cytokines (Figure 8F). These results collectively indicate that FAP, through its interaction with PLAUR, promotes the assembly of the PLAUR‐ITGB1 complex and subsequent activation of the ITGB1/FAK axis, leading to downstream NF‐κB and MAPK signaling and enhanced pro‐inflammatory cytokine production in macrophages.

### 3.8 The ITGB1/FAK Pathway Mediates FAP‐Regulated TAD Pathogenesis

**FIGURE 9:**
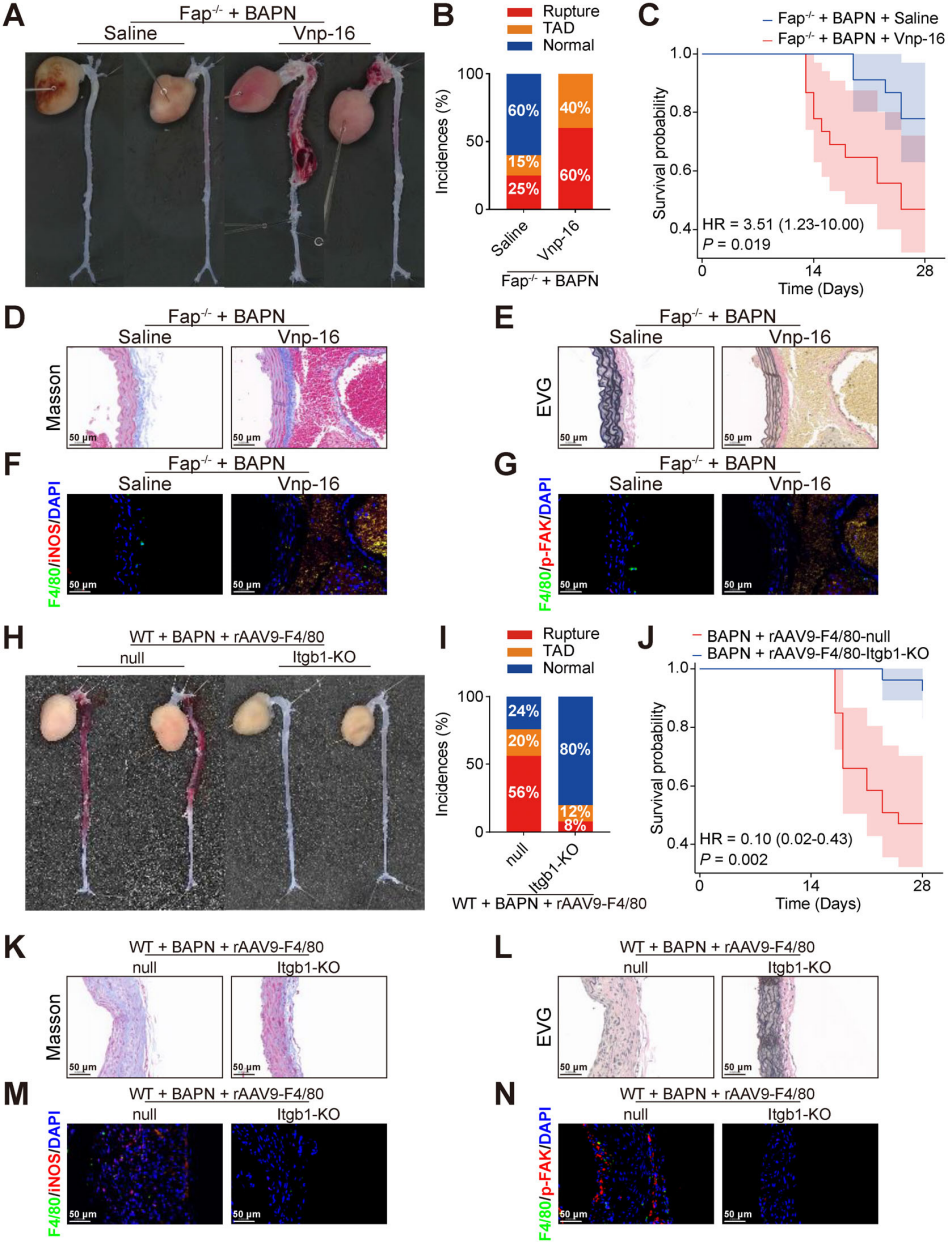
The ITGB1/FAK pathway mediates FAP‐regulated TAD pathogenesis. A) Representative macroscopic images of aortas from Fap^−/−^ mice treated with saline or Vnp‐16 following 28‐day BAPN administration. B) Stacked bar chart showing the incidence of normal aorta (blue), TAD (orange), and rupture (red) in the two groups (*n* = 20 per group). C) Survival probability between the Saline and Vnp‐16 groups was compared by Cox proportional hazards model (*n* = 20 per group). D,E) Representative histological staining (Masson [C], EVG [D]) of aortic sections between the Saline and Vnp‐16 groups after 28‐day BAPN administration. Scale bars, 50 µm. F) Immunofluorescence staining for F4/80 (green) and iNOS (red), with DAPI (blue) in aortic samples from the Saline and Vnp‐16 groups. Scale bars, 50 µm. G) Immunofluorescence staining for F4/80 (green) and phosphorylated FAK (red), with DAPI (blue) in aortic samples from the Saline and Vnp‐16 groups. Scale bars, 50 µm. H) Representative macroscopic images of aortas from rAAV‐F4/80‐Itgb1‐KO or rAAV‐F4/80‐null groups following 28‐day BAPN administration. I) Stacked bar chart showing the incidence of normal aorta (blue), TAD (orange), and rupture (red) in the two groups (*n* = 25 per group). J) Survival probability between the rAAV‐F4/80‐Itgb1‐KO or rAAV‐F4/80‐null groups was compared by Cox proportional hazards model (*n* = 25 per group). K,L) Representative histological staining (Masson [C], EVG [D]) of aortic sections between rAAV‐F4/80‐Itgb1‐KO and rAAV‐F4/80‐null groups after 28 days of BAPN administration. Scale bars, 50 µm. M) Immunofluorescence staining for F4/80 (green) and iNOS (red), with DAPI (blue) in aortic samples from the rAAV‐F4/80‐Itgb1‐KO or rAAV‐F4/80‐null group. Scale bars, 50 µm. N) Immunofluorescence staining for F4/80 (green) and phosphorylated FAK (red), with DAPI (blue) in aortic samples from rAAV‐F4/80‐Itgb1‐KO or rAAV‐F4/80‐null group. Scale bars, 50 µm.

Our previous findings demonstrated that FAP/PLAUR interaction activated the ITGB1/FAK signaling cascade, thereby enhancing macrophage pro‐inflammatory polarization (Figures 6, 7, 8). However, whether ITGB1/FAK serves as the critical downstream effector of FAP/PLAUR in TAD pathogenesis remains to be experimentally validated. It is reported that vitronectin‐derived peptide Vnp‐16 exerts its biological activity by directly interacting with ITGB1 and subsequently activating FAK signaling [16]. In this study, we employed Vnp‐16 to investigate the role of ITGB1/FAK activation in TAD development. Histological and biochemical analyses in mice with Vnp‐16 revealed no significant differences compared with the saline‐treated control group (Figure S19). Fap^−/−^ mice treated with saline or Vnp‐16 were then utilized to establish the TAD models through 28‐day BAPN administration (*n* = 20 per group). The results showed that Vnp‐16 supplementation reversed the protective effect conferred by Fap deletion in TAD development (Figures 9A–E and S20A,B). Immunofluorescence analysis of aortic tissues revealed that, compared with the Fap^−/−^ mice, Vnp‐16‐mediated ITGB1 activation markedly increased iNOS and p‐FAK expression in F4/80^+^ macrophages (Figures 9F,G and S20C,D).

To further investigate whether the regulatory effect of FAP on TAD depends on the activation of ITGB1 in macrophages, we constructed an rAAV9 carrying an F4/80 promoter‐driven Itgb1 knockout construct (rAAV‐F4/80‐Itgb1‐KO, Figure S21). Following a 28‐day BAPN challenge, the rAAV‐F4/80‐Itgb1‐KO group exhibited significantly lower incidence of TAD and reduced mortality compared with the rAAV‐F4/80‐null group (*n* = 25 per group, Figures 9H–L and S20E,F). Immunofluorescence colocalization analysis of aortic tissues revealed that, compared with the rAAV‐F4/80‐null group, rAAV‐F4/80‐Itgb1‐KO group significantly reduced iNOS and p‐FAK expression in F4/80^+^ macrophages (Figures 9M,N and S20G,H). These findings suggest that the ITGB1/FAK signaling pathway may act as a potential downstream mechanistic axis mediating FAP/PLAUR interaction‐regulated TAD progression (Figure S22).

## 4 Discussion

This study investigates the biological effects and mechanisms of fibroblast‐derived FAP, a key enzyme regulating ECM remodeling, in the development of TAD. Using global and fibroblast‐specific Fap knockout mice, we demonstrated that FAP deficiency reduces TAD risk by alleviating ECM disruption and suppressing inflammatory responses. However, pharmacological inhibition of FAP's enzymatic activity failed to mitigate BAPN‐induced elastin degradation or inflammatory cell infiltration. Further single‐cell sequencing analysis, cellular and animal experiments revealed that fibroblast‐derived FAP binds to macrophage PLAUR, triggering ITGB1‐dependent FAK phosphorylation, which ultimately promotes pro‐inflammatory signaling and contributes to TAD progression. These findings collectively offer novel insights into FAP's nonenzymatic functions in TAD pathogenesis, providing a foundation for developing targeted FAP‐based therapies for TAD.

During embryonic development, FAP exhibits transient expression within somites, myotubes, and perichondrial mesenchyme, with repression occurring after birth [17]. In healthy adults, low basal FAP levels are detectable in tissues including muscle, bone marrow, adipose tissue, skin, and pancreas [18]. Under physiological conditions, FAP is highly expressed within the granulation tissue of surgical wounds, functioning as a marker of wound repair [19]. In cardiovascular pathologies, FAP is upregulated within SMCs of rupture‐prone thin‐cap atheromas [6]. Expression peaks around day 7 in peri‐infarct myofibroblasts following myocardial infarction, while circulating soluble FAP levels inversely correlate with myocardial damage severity [20]. Cardiac FAP expression increased in patients with heart failure (HF) or hypertrophic cardiomyopathy, correlating with fibrosis [21]. In our study, FAP was specifically expressed in aortic fibroblasts and demonstrates significant upregulation in both human TAD and mouse TAD models. Existing research has reported significant upregulation of FAP in AAA, detectable via PET‐CT imaging [7]. These findings highlight the need for less invasive methods to detect FAP, which could improve prediction and monitoring of TAD progression. In contrast to pathologies where FAP acts primarily through proteolytic activity, our results demonstrate that a nonenzymatic mechanism predominates in TAD pathogenesis. This process entails fibroblast‐macrophage communication via the FAP/PLAUR/ITGB1 axis, differentiating its function in TAD from that in other cardiovascular and fibrotic disorders.

The DPP family comprises enzymatically active members such as DPP4, DPP8, DPP9, and FAP [22]. These enzymes hydrolyze prolyl bonds, releasing dipeptides from protein N‐termini. The study by Lu et al. [11] revealed that increased plasma DPP4 activity correlated with AAA growth in humans, while decreased membrane DPP4 expression on monocytes facilitated monocyte‐to‐macrophage differentiation via the ERK1/2‐p21 signaling pathway and cytoskeletal reorganization, contributing to aneurysm progression. Bao et al. [23] found that orally administered alogliptin, a DPP‐4 inhibitor, dose‐dependently attenuated AAA formation and expansion in a rat model. FAP shares approximately 70% amino acid sequence identity with DPP4 and exhibits DPP activity [24]. Nevertheless, FAP uniquely exhibits endopeptidase activity, a distinct characteristic leading to speculation that this constitutes its predominant and unique enzymatic role [25]. In HF, activated cardiac fibroblasts highly express FAP, which degrades brain natriuretic peptide (BNP) and suppresses its pro‐angiogenic activity, thereby impeding cardiac repair; conversely, FAP inhibition stabilizes BNP levels and activates the BNP/Npr1/cGMP signaling pathway, promoting angiogenesis, enhancing organized ECM deposition, and restricting fibrotic scar expansion to improve cardiac function [21]. In aortic atherosclerotic plaques, FAP expressed by plaque fibroblasts and SMCs disrupts ECM structure by cleaving collagen (particularly pro‐COL3A1) [26]. Current evidence indicates FAP exerts regulatory control over the ECM in the cardiovascular system through its endopeptidase activity. However, our findings revealed no protective effect of the FAP protease inhibitor on TAD, suggesting that FAP may employ alternative mechanisms to regulate ECM, consequently necessitating further exploration.

Beyond its enzymatic activity, FAP plays a significant role in regulating intracellular signaling pathways [27]. Studies utilizing catalytically inactive FAP mutants (Ser642Ala) demonstrate functional impacts independent of proteolytic function [28]. Mechanistically, FAP localizes to invadopodia via integrin α3β1 and associates with the PLAUR signaling complex, mediating ECM degradation and invasion [29]. FAP expression activates the PI3K/AKT and SHH/Gli1 pathways, promoting cell cycle progression [30, 31]. Furthermore, FAP contributes to immunosuppression by promoting CCL2 expression via a PLAUR‐dependent FAK‐Src‐STAT3 pathway, where STAT3 acts as the transcription factor [15]. FAP promotes non‐small cell lung cancer metastasis by activating the integrin/FAK signaling pathway through its interaction with integrin α3β1, leading to enhanced cell migration and invasion [32]. In our study, fibroblast‐secreted FAP interacts with macrophage PLAUR, thereby activating the ITGB1/FAK signaling pathway and accelerating the progression of TAD. These findings suggest that FAP‐regulated intracellular signaling pathways play a crucial role in vascular ECM modulation, warranting further investigation.

The advancement of single‐cell sequencing has progressively revealed the modulatory roles of fibroblast‐macrophage interactions within the ECM and the immune microenvironment. Yuan et al. [33] revealed a pathological niche of colorectal cancer where FAP^+^ fibroblasts and SPP1^+^ macrophages interact via chemerin, TGF‐β, and IL‐1 signaling to drive desmoplasia and immune exclusion, contributing to immune checkpoint blockade resistance [33]. Amrute et al. [34] utilized multiomic and spatial technologies to define a pathogenic FAP/POSTN fibroblast lineage in HF, with immunostaining confirming co‐localization of FAP^+^ fibroblasts and CCR2^+^ macrophages. Our analysis of normal and TAD aortic tissues revealed significantly upregulated communication between FAP^+^ fibroblasts and IL1B^+^ macrophages in TAD. Pro‐inflammatory macrophages, recruited to the arterial wall, directly degrade ECM components such as elastin by secreting matrix metalloproteinases (MMPs), including MMP‐2 and MMP‐9 [35]. This degradation is amplified through the release of inflammatory cytokines like TNF‐α and IL‐1β, which enhance MMP activity and sustain a destructive inflammatory environment [36]. Our study demonstrates that FAP promotes a pro‐inflammatory macrophage phenotype by interacting with the PLAUR, leading to subsequent activation of the ITGB1/FAK signaling pathway. Evidence from the literature and our findings suggest that the intercellular communication between FAP^+^ fibroblasts and IL1B^+^ macrophages plays a critical role in the pathogenesis of TAD, highlighting it as a potential therapeutic target.

There were several limitations to this study. (1) Given the extensive biological effects of FAP^+^ fibroblasts, the possibility of their crosstalk with SMCs or ECs cannot be ruled out. (2) While our study focused on the direct regulatory role of FAP in macrophages, it is important to note that FAP is a pleiotropic protein expressed in multiple cell types (e.g., SMCs and ECs) and may therefore also shape the TAD microenvironment through indirect mechanisms. (3) The overall pharmacokinetic profile of FAP inhibitors warrants further exploration in subsequent studies.

## 5 Conclusion

Fibroblast‐derived FAP promotes TAD progression through a nonenzymatic mechanism involving its direct interaction with PLAUR expressed on macrophages. This interaction activates ITGB1/FAK signaling, thereby facilitating pro‐inflammatory macrophage polarization. Targeted disruption of the FAP/PLAUR interaction may offer a promising therapeutic strategy to attenuate inflammatory aortic remodeling and prevent TAD development.

## Author Contributions

Z.P.J., J.Z., R.F. together with J.J.L. conceptually designed the study. J.L.W. and H.Q.Z. conducted clinical sample collection, animal experiment, cell experiment, and draft writing. Z.Y.X. and Y.F.P. assisted in clinical sample collection and data analysis. Z.P.J., J.Z., R.F. and J.J.L. provided reagents, conceptual advice and critically reviewed the manuscript.

## Funding

The project was supported by National Natural Science Foundation of China (81900418, J.J.L., 82270505, R.F.) and Qingdao Municipal Bureau of Science and Technology, Natural Science Foundation of Qingdao Municipality (23‐2‐1‐194‐zyyd‐jch, J.J.L.).

## Conflicts of Interest

The authors declare no conflicts of interest.

## Supporting information




**Supporting File**: advs74358‐sup‐0001‐SuppMat.docx.

## Data Availability

The data that support the findings of this study are available from the corresponding author upon reasonable request.

## References

[1] L. Mazzolai , G. Teixido‐Tura , S. Lanzi , et al., “2024 ESC Guidelines for the Management of Peripheral Arterial and Aortic Diseases,” European Heart Journal 45 (2024): 3538–3700, 10.1093/eurheartj/ehae179. 39210722

[2] C. A. Nienaber and X. Yuan , “Taming Hypertension to Prevent Aortic Dissection: Universal Recognition of a ‘New Normal’ Blood Pressure?,” Circulation 145 (2022): 645–647, 10.1161/CIRCULATIONAHA.121.058133. 35226554

[3] X. Kou , X. Xu , C. Chen , et al., “The Fas/Fap‐1/Cav‐1 Complex Regulates IL‐1RA Secretion in Mesenchymal Stem Cells to Accelerate Wound Healing,” Science Translational Medicine 10 (2018): aai8524, 10.1126/scitranslmed.aai8524. PMC6310133 29540618

[4] L. Kotacková , E. Baláziová , and A. Sedo , “Expression Pattern of Dipeptidyl Peptidase IV Activity and/or Structure Homologues in Cancer,” Folia Biologica 55 (2009): 77–84, 10.14712/fb2009055030077. 19545486

[5] F. M. Keane , T.‐W. Yao , S. Seelk , et al., “Quantitation of Fibroblast Activation Protein (FAP)‐Specific Protease Activity in Mouse, Baboon and Human Fluids and Organs,” FEBS Open Bio 4 (2013): 43–54, 10.1016/j.fob.2013.12.001. PMC3871272 24371721

[6] C. E. Brokopp , R. Schoenauer , P. Richards , et al., “Fibroblast Activation Protein Is Induced by Inflammation and Degrades Type I Collagen in Thin‐cap Fibroatheromata,” European Heart Journal 32 (2011): 2713–2722, 10.1093/eurheartj/ehq519. 21292680 PMC3205479

[7] C. Hu , H. Tan , Y. Zhang , et al., “Fibroblast Activation Protein Acts as a Biomarker for Monitoring ECM Remodeling during Aortic Aneurysm via^68^ Ga‐FAPI‐04 PET Imaging,” Advanced Science 12 (2025): 2411152, 10.1002/advs.202411152. 39950910 PMC11984865

[8] J. Barallobre‐Barreiro , B. Loeys , M. Mayr , M. Rienks , A. Verstraeten , and J. C. Kovacic , “Extracellular Matrix in Vascular Disease, Part 2/4,” Journal of the American College of Cardiology 75 (2020): 2189–2203, 10.1016/j.jacc.2020.03.018. 32354385

[9] V. J. Christiansen , K. W. Jackson , K. N. Lee , and P. A. McKee , “Effect of Fibroblast Activation Protein and α2‐antiplasmin Cleaving Enzyme on Collagen Types I, III, and IV,” Archives of Biochemistry and Biophysics 457 (2007): 177–186, 10.1016/j.abb.2006.11.006. 17174263 PMC1857293

[10] A. A. Fitzgerald and L. M. Weiner , “The Role of Fibroblast Activation Protein in Health and Malignancy,” Cancer and Metastasis Reviews 39 (2020): 783–803, 10.1007/s10555-020-09909-3. 32601975 PMC7487063

[11] H.‐Y. Lu , C.‐Y. Huang , C.‐M. Shih , et al., “A Potential Contribution of Dipeptidyl Peptidase‐4 by the Mediation of Monocyte Differentiation in the Development and Progression of Abdominal Aortic Aneurysms,” Journal of Vascular Surgery 66 (2017): 1217–1226.e1, 10.1016/j.jvs.2016.05.093. 27887857

[12] C. Y. Edosada , C. Quan , C. Wiesmann , et al., “Selective Inhibition of Fibroblast Activation Protein Protease Based on Dipeptide Substrate Specificity,” Journal of Biological Chemistry 281 (2006): 7437–7444, 10.1074/jbc.M511112200. 16410248

[13] H. Wei , Y. Xu , Y. Wang , et al., “Identification of Fibroblast Activation Protein as an Osteogenic Suppressor and Anti‐osteoporosis Drug Target,” Cell Reports 33 (2020): 108252, 10.1016/j.celrep.2020.108252. 33053358

[14] S. M. Pokharel , N. K. Shil , J. B. GC , et al., “Integrin Activation by the Lipid Molecule 25‐hydroxycholesterol Induces a Proinflammatory Response,” Nature Communications 10 (2019): 1482, 10.1038/s41467-019-09453-x. PMC6443809 30931941

[15] X. Yang , Y. Lin , Y. Shi , et al., “FAP Promotes Immunosuppression by Cancer‐Associated Fibroblasts in the Tumor Microenvironment via STAT3–CCL2 Signaling,” Cancer Research 76 (2016): 4124–4135, 10.1158/0008-5472.CAN-15-2973. 27216177

[16] S.‐K. Min , H. K. Kang , S. Y. Jung , D. H. Jang , and B.‐M. Min , “A Vitronectin‐Derived Peptide Reverses Ovariectomy‐Induced Bone Loss via Regulation of Osteoblast and Osteoclast Differentiation,” Cell Death & Differentiation 25 (2018): 268–281, 10.1038/cdd.2017.153. 28937683 PMC5762842

[17] J. Niedermeyer , P. Garin‐Chesa , M. Kriz , et al., “Expression of the Fibroblast Activation Protein During Mouse Embryo Development,” The International Journal of Developmental Biology 45 (2001): 445–447, 10.1387/ijdb.11330865. 11330865

[18] E. W. Roberts , A. Deonarine , J. O. Jones , et al., “Depletion of Stromal Cells Expressing Fibroblast Activation Protein‐α From Skeletal Muscle and Bone Marrow Results in Cachexia and Anemia,” Journal of Experimental Medicine 210 (2013): 1137–1151, 10.1084/jem.20122344. 23712428 PMC3674708

[19] K. M. McAndrews , T. Miyake , E. A. Ehsanipour , et al., “Dermal αSMA+ Myofibroblasts Orchestrate Skin Wound Repair via β1 Integrin and Independent of Type I Collagen Production,” The EMBO Journal 41 (2022): 109470, 10.15252/embj.2021109470. PMC8982612 35212000

[20] J. Tillmanns , D. Hoffmann , Y. Habbaba , et al., “Fibroblast Activation Protein Alpha Expression Identifies Activated Fibroblasts After Myocardial Infarction,” Journal of Molecular and Cellular Cardiology 87 (2015): 194–203, 10.1016/j.yjmcc.2015.08.016. 26319660

[21] Y. Sun , M. Ma , D. Cao , et al., “Inhibition of Fap Promotes Cardiac Repair by Stabilizing BNP,” Circulation Research 132 (2023): 586–600, 10.1161/CIRCRESAHA.122.320781. 36756875

[22] K. N. Lee , K. W. Jackson , V. J. Christiansen , C. S. Lee , J.‐G. Chun , and P. A. McKee , “Antiplasmin‐Cleaving Enzyme Is a Soluble Form of Fibroblast Activation Protein,” Blood 107 (2006): 1397–1404, 10.1182/blood-2005-08-3452. 16223769

[23] W. Bao , K. Morimoto , T. Hasegawa , et al., “Orally Administered Dipeptidyl Peptidase‐4 Inhibitor (alogliptin) Prevents Abdominal Aortic Aneurysm Formation Through an Antioxidant Effect in Rats,” Journal of Vascular Surgery 59 (2014): 1098–1108, 10.1016/j.jvs.2013.04.048. 23790558

[24] K. Aertgeerts , I. Levin , L. Shi , et al., “Structural and Kinetic Analysis of the Substrate Specificity of Human Fibroblast Activation Protein α,” Journal of Biological Chemistry 280 (2005): 19441–19444, 10.1074/jbc.C500092200. 15809306

[25] K. N. Lee , K. W. Jackson , S. Terzyan , V. J. Christiansen , and P. A. McKee , “Using Substrate Specificity of Antiplasmin‐Cleaving Enzyme for Fibroblast Activation Protein Inhibitor Design,” Biochemistry 48 (2009): 5149–5158, 10.1021/bi900257m. 19402713 PMC4470291

[26] S. Stein , J. Weber , S. Nusser‐Stein , et al., “Deletion of Fibroblast Activation Protein Provides Atheroprotection,” Cardiovascular Research 117 (2021): 1060–1069, 10.1093/cvr/cvaa142. 32402085

[27] H. Wang , Q. Wu , Z. Liu , et al., “Downregulation of FAP Suppresses Cell Proliferation and Metastasis Through PTEN/PI3K/AKT and Ras‐ERK Signaling in Oral Squamous Cell Carcinoma,” Cell death & disease 5 (1155), 10.1038/cddis.2014.122. PMC5424105 24722280

[28] T. Ramirez‐Montagut , N. E. Blachere , E. V. Sviderskaya , et al., “FAPα, a Surface Peptidase Expressed During Wound Healing, Is a Tumor Suppressor,” Oncogene 23 (2004): 5435–5446, 10.1038/sj.onc.1207730. 15133496

[29] V. V. Artym , A. L. Kindzelskii , W.‐T. Chen , and H. R. Petty , “Molecular Proximity of Seprase and the Urokinase‐Type Plasminogen Activator Receptor on Malignant Melanoma Cell Membranes: Dependence on beta1 Integrins and the Cytoskeleton,” Carcinogenesis 23 (2002): 1593–1602, 10.1093/carcin/23.10.1593. 12376466

[30] J. Jia , T. A. Martin , L. Ye , et al., “Fibroblast Activation Protein‐α Promotes the Growth and Migration of Lung Cancer Cells via the PI3K and Sonic Hedgehog Pathways,” International Journal of Molecular Medicine 41 (2018): 275–283, 10.3892/ijmm.2017.3224. 29115573 PMC5746330

[31] D. Yue , H. Li , J. Che , et al., “Hedgehog/Gli Promotes Epithelial‐Mesenchymal Transition in Lung Squamous Cell Carcinomas,” Journal of Experimental & Clinical Cancer Research 33 (2014): 34, 10.1186/1756-9966-33-34. 24758269 PMC4029998

[32] L. Gao , A. Wang , Y. Chen , et al., “FTO Facilitates Cancer Metastasis by Modifying the m6A Level of FAP to Induce Integrin/FAK Signaling in Non‐small Cell Lung Cancer,” Cell Communication and Signaling 21 (2023): 311, 10.1186/s12964-023-01343-6. 37919739 PMC10623768

[33] Z. Yuan , H. Hu , Y. Zhu , et al., “Colorectal Cancer Cell Intrinsic Fibroblast Activation Protein Alpha Binds to Enolase1 and Activates NF‐κB Pathway to Promote Metastasis,” Cell Death & Disease 12 (2021): 543, 10.1038/s41419-021-03823-4. 34035230 PMC8149633

[34] J. M. Amrute , X. Luo , V. Penna , et al., “Targeting Immune–Fibroblast Cell Communication in Heart Failure,” Nature 635 (2024): 423–433, 10.1038/s41586-024-08008-5. 39443792 PMC12334188

[35] M. Vandestienne , Y. Zhang , I. Santos‐Zas , et al., “TREM‐1 Orchestrates Angiotensin II–Induced Monocyte Trafficking and Promotes Experimental Abdominal Aortic Aneurysm,” Journal of Clinical Investigation 131 (2021): 142468, 10.1172/JCI142468. 33258804 PMC7810476

[36] J. L. Eliason , K. K. Hannawa , G. Ailawadi , et al., “Neutrophil Depletion Inhibits Experimental Abdominal Aortic Aneurysm Formation,” Circulation 112 (2005): 232–240, 10.1161/CIRCULATIONAHA.104.517391. 16009808

