# Supplementary material for: Fibroblast Activation Protein Promotes Thoracic Aortic Dissection via PLAUR/ITGB1‐Mediated Pro‐inflammatory Macrophage Polarization
Source: Adv Sci (Weinh). 2026 Feb 12;13(23):e14786. doi: 10.1002/advs.202514786 (PMC13104128; doi:10.1002/advs.202514786)
Supplement: Supplementary file 1 — Supporting File: advs74358‐sup‐0001‐SuppMat.docx. [file ADVS-13-e14786-s001.docx]

**SUPPLEMENTAL MATERIAL**

**Title:** Fibroblast activation protein promotes thoracic aortic dissection via PLAUR/ITGB1-mediated pro-inflammatory macrophage polarization

**Authors:** Hongqiao Zhu^1#^, Jianlie Wu^2#^, Ziyi Xu^1^, Yifei Pei^3^, Zaiping Jing^3^, Jian Zhou^4^, Rui Feng^1*^, Junjun Liu^1*^.

^1^Department of Vascular Surgery, Shanghai General Hospital, Shanghai Jiao Tong University School of Medicine, Shanghai, China.

^2^ Department of Nursing, Shanghai General Hospital, Shanghai Jiao Tong University School of Medicine, Shanghai, China.

^3^Department of Vascular Surgery, Shanghai Fourth People’s Hospital, School of Medicine, Tongji University, Shanghai, China.

^4^Department of Vascular Surgery, The First Affiliated Hospital, Naval Medical University, Shanghai, China.

*Corresponding authors:

Professor Rui Feng, 650 Xinsongjiang Road, Songjiang District, Shanghai, China, email: webmaster@xueguan.net

Professor Junjun Liu, 650 Xinsongjiang Road, Songjiang District, Shanghai, China, email: Junjun.liu@shgh.cn

**Supplementary information:**

-Supplementary methods

-Supplementary figures 1 to 22

-Supplementary tables 1 to 4

**Supplemental materials and methods**

**1. Animal models**

**1.1 TAD induction:** Male wild-type (WT) mice (3 weeks old) received 0.25% (wt/vol) β-aminopropionitrile (BAPN, #A3134, Sigma, USA) in drinking water for 28 days^1^.

**1.2 Genetic models:** Global Fap knockout (Fap^-/-^, S-KO-02005, Cyagen Biosciences Inc., China) and fibroblast-specific Fap knockout (Fap-flox [S-CKO-02356] × Postn-P2A-iCre [I001166], Cyagen Biosciences Inc., China) mice were generated on a C57BL/6J background.

**1.3 Pharmacological inhibition of FAP:** WT mice received daily oral gavage of Ac-Gly-BoroPro (50 μg/kg or 500 μg/kg; MedChemExpress, #HY-101801) or saline, starting concurrently with BAPN.

**1.4 ITGB1 Activation:** Fap^-/-^ mice were treated with Vnp-16 (1 mg/kg; MedChemExpress, #HY-P5929) via intraperitoneal injection once every three days. In the in vitro experiments, macrophages in different experimental groups were treated with Vnp-16 at a concentration of 0.1 mg/ml.

**1.5 In vivo transduction:** Recombinant adeno-associated virus serotype 9 (rAAV9) carrying a C-terminal Flag-tagged Postn-promoter-driven construct (null, FAP, FAP^S624A^, or FAP^mutant^) was obtained from HanBio, Shanghai (2.1 × 10^12^ vg/ml). Additionally, rAAV9 expressing a ZsGreen-tagged F4/80-promoter-driven construct (null, mir30-1-itgb1 [ITGB1-KO#1], or mir30-2-itgb1 [ITGB1-KO#2]) was also sourced from HanBio, Shanghai (2.0 × 10^12^ vg/ml). Each viral preparation (100 μl) was administered via tail vein injection to mice at 18 days of age. Seven days later, TAD was induced by supplementing the drinking water with 0.25% (wt/vol) BAPN for a duration of four weeks.

**1.6 Tissue harvest:** Daily monitoring was conducted to assess the aortic-related events. At the experimental endpoint, mice were euthanized by intraperitoneal injection of pentobarbital sodium (200 mg/kg), and tissues were harvested for histological examination. For arterial tissues intended for RNA/protein extraction and cell isolation, cardiac perfusion with ice-cold phosphate-buffered saline (PBS) was performed; those designated for histological and immunofluorescence staining were perfused with PBS followed by fixation in 4% paraformaldehyde.

**2. Cell isolation, culture, and transfection​**​

**2.1 Cell isolation and culture:** The isolation of fibroblasts, endothelial cells, and smooth muscle cells (SMCs) from murine aortas was performed according to established methodologies^2^. Fibroblasts, endothelial cells and SMCs isolated via collagenase digestion from aortas of both WT and Fap^-/-^ mice were cultured in DMEM (Dulbecco’s Modified Eagle’s Medium, PM150210, Procell, China) supplemented with 10% FBS (Fetal Bovine Serum, 164220, Procell, China). Bone marrow-derived macrophages (BMDMs) were obtained by flushing the bone marrow from both the femur and tibia of mice according to previous study^3^. The harvested cells were then cultured in DMEM supplemented with 10% FBS. They were all maintained in a temperature-regulated incubator at 37°C with a 5% CO_2_ atmosphere in a humidified setting (at a level of approximately 85%).

**2.2 Cell co-culture:** A co-culture system for aortic fibroblasts and BMDMs was set up using a 24-well culture insert with 0.4 μm pores (CLS3415, Corning, USA), where the fibroblasts were seeded in the upper compartment and BMDMs in the lower compartment of a 24-well plate.

**2.3 Transfections:** Double-stranded siRNAs of negative control, and Plaur were synthesized by GenePharma Biotech (Shanghai, China) (Table S1). Macrophages were transfected with siRNA using Lipo6000 Transfection Reagent (C0526, Beyotime, China).

**3. Protein extraction, immunoprecipitation, and western blotting​**​

Whole-cell lysates were prepared by resuspending cell pellets in RIPA buffer (P0013B, Beyotime, China) supplemented with fresh protease inhibitor cocktail (P1005, Beyotime, China). Cell lysates were incubated overnight with Anti-Flag Magnetic Beads (P2115, Beyotime, China) or Anti-Myc Magnetic Beads (P2118, Beyotime, China). After extensive washing, immunoprecipitated proteins were eluted and analyzed by western blotting.

Equal amounts of lysates were resolved on 10% SDS-polyacrylamide gels and transferred onto polyvinylidene fluoride membranes (TIBIO, China). Membranes were blocked with rapid blocking liquid (G2052, Servicebio, China) and probed with primary antibodies overnight at 4°C, followed by incubation with appropriate secondary antibodies. Antibodies used are listed in Table S2. Protein bands were detected using chemiluminescence detection with TanonFine-DoX6 (Tianneng, China) and quantified by densitometry (ImageJ). Protein expression levels were normalized to β-actin.

**4. Immunofluorescence staining​**​

The primary antibodies used in immunofluorescence staining were shown in Table S2. Aortic and cell samples were fixed for 15 min in 4% paraformaldehyde and subsequently permeabilized for 20 min utilizing 0.1% Triton X100. After the addition of 5% bovine serum albumin and incubation at 37°C for 1 h, the incubated cells were subsequently exposed to the primary antibody during an overnight incubation at 4°C. Following three rinses with PBS, the cells were then exposed to the secondary antibodies for 1 h at 37°C under the light-proof condition. Afterward, the cells were washed three times using PBS and incubated with DAPI (C1005, Beyotime, China). The fluorescence microscope (Leica, Germany) was used to visualize the images. Pearson’s correlation coefficients were determined from the images by using the ImageJ coloc2 plugin.

**5. Pathological staining**

Aortic tissue sections were subjected to Masson’s trichrome and Elastic van Gieson (EVG) staining to evaluate structural alterations in the extracellular matrix. Masson’s staining (Sigma-Aldrich, St. Louis, MO) was used to detect collagen deposition, where collagen fibers appear blue against a background of red-stained cytoplasm and black nuclei. The relative extent of fibrosis was quantified as the percentage of the blue-stained collagen area relative to the total aortic area^4^. EVG staining highlighted elastic fibers in blue-black, allowing for the assessment of elastin integrity. Elastin degradation was quantified by counting the number of breaks in the elastic fibers per section^4^. For each mouse, a series of 6-9 sections were examined to ensure statistical robustness. After euthanasia, mice treated with either saline or Vnp-16 underwent transcardial perfusion, followed by fixation using 4% paraformaldehyde. Tissue samples from the heart, liver, spleen, lung, and kidney were then collected, processed, and stained with hematoxylin and eosin for histological examination.

**6. Molecular dynamics**

We determined the three-dimensional structure of the mouse plasminogen activator urokinase receptor (PLAUR)-FAP protein complex through *de novo* prediction using the AlphaFold3 algorithm (version updated May 2024). Among all generated models, the conformation with the highest confidence scores (pLDDT and PAE) was selected, as it exhibited the most plausible protein folding and complex interface. This high-accuracy model served as the initial structural template for all subsequent computational simulations. All molecular dynamics (MD) simulations were performed using the GROMACS software package 4.6.5. The predicted complex was solvated in an octahedral water box. The Amber ff14SB force field was applied to the protein, and water molecules were described using the TIP3P model. The system was neutralized and brought to a physiological ion concentration of 0.15 M using Na^+^ and Cl^-^ ions.

The simulation protocol comprised three stages. First, energy minimization was conducted via the steepest descent algorithm to eliminate unfavorable atomic clashes. Subsequently, the system was gradually equilibrated under NVT (constant number of particles, volume, and temperature) and NPT (constant number of particles, pressure, and temperature) ensembles, stabilizing temperature at 300 K and pressure at 1 bar. Finally, a 100 ns production MD simulation was performed without restraints, during which trajectory data were saved every 20 ps for subsequent analysis.

The resulting 100 ns trajectory was subjected to comprehensive post-processing. Using built-in GROMACS tools and custom scripts, we calculated key structural parameters—including root-mean-square deviation (RMSD), root-mean-square fluctuation (RMSF), and radius of gyration (Rg)—to assess the overall stability, local flexibility, and structural compactness of the complex. Principal component analysis (PCA) was employed to investigate dominant collective motions. To quantitatively evaluate binding affinity, the molecular mechanics/generalized Born surface area (MM-GBSA) method was applied using the gmx_MMPBSA tool. The binding free energy was decomposed per-residue to identify key “hotspot” regions contributing to the interaction. All structural visualizations and data plotting were performed using UCSF ChimeraX.

**7. Protein expression and surface plasmon resonance (SPR) assays**

Recombinant WT proteins of mouse FAP and PLAUR were purchased from Acro Biosystems (FAP-M52H3, Beijing, China) and Sino Biological (50160-M08H-UE， Beijing, China), respectively. The FAP^S624A^ and FAP^mutant^ proteins (Table S3) were expressed in HEK293 cells incubated at 37°C under 5% CO_2_ with continuous shaking at 120 rpm for 6 days post-transfection. The culture supernatant was harvested by centrifugation at 3000g, filtered through a 0.22 μm membrane, and purified using Ni-NTA agarose resin pre-equilibrated with PBS buffer (pH 7.4), followed by stepwise elution with imidazole concentrations ranging from 30 mM to 300 mM. For SPR analysis, the Biacore 1K+ system with CM5 sensor chips was utilized; recombinant PLAUR was immobilized as the ligand via amine coupling at approximately 2040 RU, and recombinant FAP was injected as the analyte in a serial dilution (0–1000 nM) using HBSEP^+^ buffer at a flow rate of 30 μL/min. The binding data were collected over 180 s association and 300 s dissociation phases, and the kinetics were evaluated with the Biacore Insight Evaluation Software to determine affinity parameters.

**8. Real-time quantitative reverse transcription polymerase chain reaction (RT-qPCR)​**​

Trizol reagent (Tokyo, Japan) and RNeasy Plus Micro Kit (Hilden, Germany) were utilized to extract the RNA of aortic tissue and macrophages, respectively. Reverse transcription utilized Takara Bio’s kit, with qPCR performed via SYBR Green on an ABI PRISM 7900. Expression was calculated utilizing the 2−ΔΔCt method, normalized to β-actin, with triplicate assays (Table S4).

**9. Enzyme-linked immunosorbent assay (ELISA)**

ELISA detection kits of FAP (RK13580, ABclonal), IL-6 (E-EL-M0044, Elabscience), TNF-α (E-EL-M3063, Elabscience), and IL-1β (E-EL-M0037, Elabscience) were used. In brief, 48-well plates were prepared by coating each well with 100μL of the capture antibody solution prepared in coating buffer. Following an overnight incubation at 4°C, the wells were sequentially treated with biotin-conjugated antibody solution, HRP-avidin solution, TMB substrate solution, and finally, the reaction was terminated with a stop solution. The absorbance was read at 450nm within 5 min of adding the stop solution, and the results were analyzed. FAP protease activity in mouse aortic tissues was quantified using an FAP Fluorogenic Assay Kit (BPS Bioscience, San Diego, CA, USA, BPQ-80210).

**10. Bulk and single-cell RNA sequencing​​**

**10.1 Bulk RNA sequencing:** Fibroblasts from Fap^fl/fl^ and Fap^Postn^ mice and BMDMs were sequenced (Illumina NovaSeq). DEGs were identified with DESeq2 (|logFC| > 1, adj. p < 0.05).

**10.2 Single-cell RNA sequencing:** Human/murine aortic single-cell data (GSE213740, GSE222318, PRJCA003113) were processed using Seurat. The dataset GSE213740 comprises single-cell RNA sequencing data from aortic tissues of 6 patients with sporadic type A aortic dissection and 3 healthy donors, processed using the 10X Genomics platform (San Francisco, CA, USA). The dataset GSE222318 includes single-cell RNA sequencing data from 4 acute TAD, 3 subacute TAD, and 2 chronic TAD patients, along with 5 healthy donors, processed via 10X Genomics (San Francisco, CA, USA). Data from PRJCA003113 were obtained from the ascending aorta and aortic arch of mice treated with 0.5 g/kg/day BAPN or saline for 7, 14, and 21 days. For each time point, tissues from six mice were pooled for single-cell RNA sequencing using the BD Rhapsody system (BD Biosciences, USA). Fibroblast subtypes and macrophage clusters were annotated. Cell-cell communication was analyzed with CellChat.

**Reference**

1. Cui, H., Chen, Y., Li, K., Zhan, R., Zhao, M., Xu, Y., Lin, Z., Fu, Y., He, Q., Tang, P.C., et al. (2021). Untargeted metabolomics identifies succinate as a biomarker and therapeutic target in aortic aneurysm and dissection. Eur. Heart J. *42*, 4373–4385. https://doi.org/10.1093/eurheartj/ehab605.

2. Chen, J., Zhuang, R., Cheng, H.S., Jamaiyar, A., Assa, C., McCoy, M., Rawal, S., Pérez-Cremades, D., and Feinberg, M.W. (2022). Isolation and culture of murine aortic cells and RNA isolation of aortic intima and media: Rapid and optimized approaches for atherosclerosis research. Atherosclerosis *347*, 39–46. https://doi.org/10.1016/j.atherosclerosis.2022.03.011.

3. Santos, G. dos, Rogel, M.R., Baker, M.A., Troken, J.R., Urich, D., Morales-Nebreda, L., Sennello, J.A., Kutuzov, M.A., Sitikov, A., Davis, J.M., et al. (2015). Vimentin regulates activation of the NLRP3 inflammasome. Nat. Commun. *6*, 6574. https://doi.org/10.1038/ncomms7574.

4. Halushka, M.K., Angelini, A., Bartoloni, G., Basso, C., Batoroeva, L., Bruneval, P., Buja, L.M., Butany, J., d’Amati, G., Fallon, J.T., et al. (2016). Consensus statement on surgical pathology of the aorta from the Society for Cardiovascular Pathology and the Association For European Cardiovascular Pathology: II. Noninflammatory degenerative diseases — nomenclature and diagnostic criteria. Cardiovasc. Pathol. *25*, 247–257. https://doi.org/10.1016/j.carpath.2016.03.002.

**Figure S1** FAP is upregulated at the site of aortic dissection lesions, correlating with inflammatory infiltration and extracellular matrix degradation.

A) Quantification of FAP protein levels in human aortic tissues from healthy donors and TAD patients (n=9 per group; Welch’s t-test).

B) Quantification of FAP protein levels in aortic tissues from mice treated with 28-day saline or BAPN (n=9 per group; Welch’s t-test).

C-D) Quantification of FAP and CD68 fluorescent intensity in human aortic tissues: normal aorta (from healthy donors), ATAD lesion, adjacent non-dissected region in ATAD, CTAD lesion, and adjacent non-dissected region in CTAD (n=6 per group; one-way ANOVA with Tukey multiple comparison test).

E-F) Quantification of collagen volume fraction and elastin degradation score in aortic tissues: normal aorta (from healthy donors), ATAD lesion, adjacent non-dissected region in ATAD, CTAD lesion, and adjacent non-dissected region in CTAD (n=6 per group; one-way ANOVA with Tukey multiple comparison test).

Data are presented as mean ± standard deviation for panels A-F.

FAP: fibroblast activation protein; BAPN: β-aminopropionitrile; NC: healthy donor; ND: non-dissected; D: dissected; ATAD: acute thoracic aortic dissection; CTAD: chronic thoracic aortic dissection.


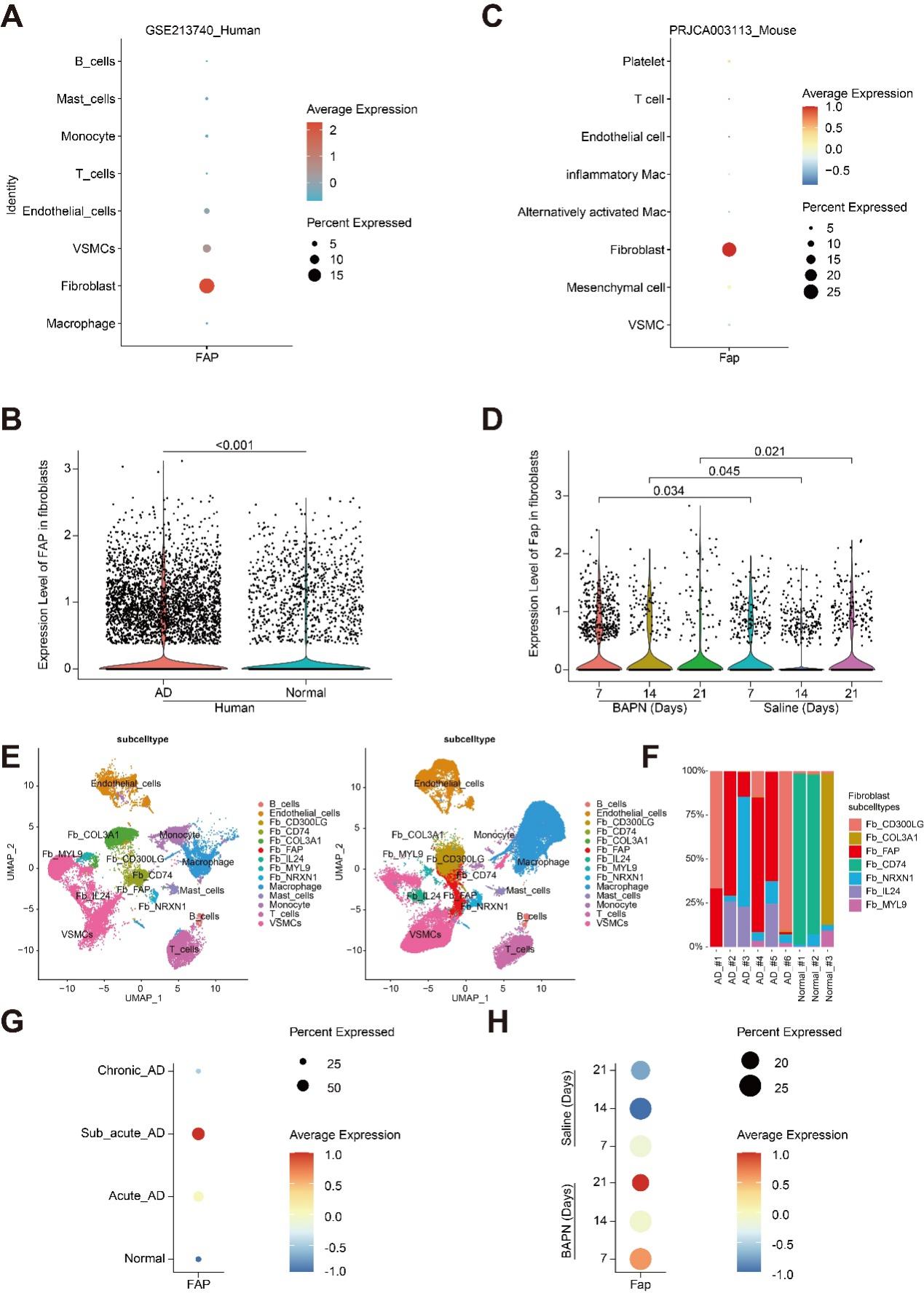


**Figure S2** Single-cell RNA sequencing reveals dynamic FAP expression in fibroblasts during TAD progression and in a BAPN-induced TAD model.

A) Dotplot depicting FAP expression across cell types in human aortic single-cell RNA-seq data (GSE213740), showing highest expression in fibroblasts.

B) Comparison of FAP expression in fibroblasts from normal donors and TAD patients from single-cell data (GSE213740; unpaired two-tailed t-test).

C) Dotplot illustrating Fap expression in mouse aortic cells (PRJCA003113), with strongest expression in fibroblasts.

D) Fap expression in fibroblasts from saline- or BAPN-treated mice across 7-, 14-, and 21-day time points from single-cell data (PRJCA003113; two-way ANOVA with Bonferroni’s multiple comparison test).

E) Uniform manifold approximation and projection visualization of 7 fibroblast subtypes in human aortic tissues from normal and TAD samples (GSE213740).

F) Stacked bar chart showing proportional changes in fibroblast subtypes between human normal and TAD aortic tissues (GSE213740).

G) Dotplot demonstrating temporal changes in fibroblast FAP expression during human TAD progression (acute, subacute, chronic phases; GSE222318).

H) Dotplot showing temporal changes in fibroblast Fap from saline- or BAPN-treated mice across 7-, 14-, and 21-day time points (PRJCA003113).

Data are presented as violin plots showing data distribution for panels B and D.

FAP: fibroblast activation protein; BAPN: β-aminopropionitrile; TAD: thoracic aortic dissection.

**Figure S3** Immunofluorescence analysis of FAP colocalization with aortic cell subtypes in human normal and acute TAD tissues.

1. C) Representative immunofluorescence images of human aortic tissue from normal donors and patient with acute TAD. Tissues were stained for FAP (red), and markers for endothelial cells (CD31, A), smooth muscle cells (αSMA, B), or macrophages (CD68, C) shown in green. Nuclei were counterstained with DAPI (blue). Quantification of colocalization using Pearson’s correlation coefficient (r). Scale bars, 100 µm.

FAP: fibroblast activation protein; TAD: thoracic aortic dissection.

**Figure S4** Expression profiling of FAP in various cell populations isolated from mice.

1. Schematic diagram of the experimental timeline and procedure. Three-week-old male mice were administered 0.25% (wt/vol) BAPN or saline for 28 days. Primary aortic fibroblasts, aortic ECs, SMCs, and BMDMs were subsequently isolated and cultured in vitro for 24 hours. The cultured cells were subjected to immunofluorescence staining to assess FAP colocalization, while the conditioned cell culture supernatants were collected for measuring secreted FAP levels by ELISA.
2. D)​ Representative immunofluorescence images showing FAP expression (red) in aortic ECs (CD31, B, green), aortic SMCs (αSMA, C, green), and BMDMs (F4/80, D, green)​ from saline- or BAPN-treated mice. Nuclei were counterstained with DAPI (blue). Quantification of colocalization using Pearson’s correlation coefficient (r). Scale bars, 100 µm.

IF: Immunofluorescence; ELISA: Enzyme-linked immunosorbent assay; ECs: endothelial cells; SMCs: smooth muscle cells; BMDMs: bone marrow-derived macrophages; FAP: fibroblast activation protein; BAPN: β-aminopropionitrile.

**Figure S5** FAP and macrophage infiltration (F4/80) are upregulated in aortic tissues during early stages of the BAPN-induced TAD model.

A-C) Quantitative analysis of the FAP fluorescent intensity in the ascending aorta (A), aortic arch (B), and descending aorta (C) from mice sacrificed at 1, 3, 5, 7, and 10 days after BAPN administration, with saline group as controls (n=9 fields per mouse; one‑way ANOVA with Tukey’s post hoc test was used for multiple comparisons).

D) Representative time‑course immunofluorescence images showing F4/80 expression (red) in aortic sections (ascending aorta, aortic arch, and descending aorta) from BAPN‑treated and control mice at indicated time points. Nuclei were counterstained with DAPI (blue). Scale bars, 50 μm.

E-G) Quantification of the F4/80 fluorescent intensity in the ascending aorta (E), aortic arch (F), and descending aorta (G) from each group (n=9 fields per mouse; one‑way ANOVA with Tukey’s test was used for statistical evaluation).

Data are presented as mean ± standard deviation for panels A-C and E-G.

FAP: fibroblast activation protein; BAPN: β-aminopropionitrile; TAD: thoracic aortic dissection.

**Figure S6** Temporal assessment of collagen deposition in aortic tissues following BAPN administration.​

A) Representative Masson’s trichrome staining of aortic sections (ascending aorta, aortic arch, and descending aorta) from mice treated with BAPN or saline at 1, 3, 5, 7, and 10 days post administration. Scale bars, 50 µm.

B-D) Quantification of relative collagen volume fraction in the ascending aorta (B), aortic arch (C), and descending aorta (D) of mice from each group (n=9 fields per mouse; one‑way ANOVA with Tukey’s test was used for statistical evaluation).

Data are presented as mean ± standard deviation for panels B-D.

BAPN: β-aminopropionitrile.

**Figure S7** Temporal assessment of aortic elastin integrity following BAPN administration.

A) Representative images of EVG aortic sections (ascending aorta, aortic arch, and descending aorta) from mice treated with BAPN or saline control at days 1, 3, 5, 7, and 10 days post administration. Scale bars, 50 µm.

B–D) Quantification of elastin degradation scores in the ascending aorta (B), aortic arch (C), and descending aorta (D) (n=9 fields per mouse; one‑way ANOVA with Tukey’s test was used for statistical evaluation).

Data are presented as mean ± standard deviation for panels B-D.

EVG: Elastic van Gieson; BAPN: β-aminopropionitrile.

**Figure S8** Genetic deletion of Fap attenuates BAPN-induced early-stage inflammation and extracellular matrix degradation.

A) PCR analysis of genomic DNA derived from WT and Fap^-/-^ mice.

B) WB analysis of aortic tissues from WT and Fap^-/-^ mice with 28-day BAPN administration (n=3 per group; two-way ANOVA with Bonferroni multiple comparison test).

C, D) Quantification of the FAP (C) and F4/80 (D) fluorescent intensity in aortas from WT and Fap^-/-^ mice under control conditions or after 5 or 10 days of BAPN treatment (n=6 fields per mouse; two-way ANOVA with Bonferroni multiple comparison test).

E, F) Quantification of the collagen volume fraction and degeneration scores in aortas from WT and Fap^-/-^ mice under control conditions or after 5 or 10 days of BAPN treatment (n=6 fields per mouse; two-way ANOVA with Bonferroni multiple comparison test).

Data are presented as mean ± standard deviation for panels B-F.

FAP: fibroblast activation protein; BAPN: β-aminopropionitrile; WB: western blot; PCR: polymerase chain reaction; WT: wild-type.

**Figure S9** Global Fap deletion attenuates inflammation and extracellular matrix degradation in a BAPN-induced TAD mouse model

A, B) Representative immunofluorescence images (A) and quantitative analysis of fluorescence intensity (B) of F4/80^+^ macrophages (red) in aortic sections from WT and Fap^-/-^ mice following 28 days of BAPN administration (n=6 fields per mouse; unpaired two-tailed t-test). Nuclei were counterstained with DAPI (blue). Scale bars, 50 µm.

C-F) Representative histological staining (Masson [C], EVG [E]) of aortic sections and quantification analysis (Masson [D], EVG [F]) between WT and Fap^-/-^ mice after 28 days of BAPN administration (n=6 field per mouse; Welch’s t-test for panel D and unpaired two-tailed t-test for panel F). Scale bars, 50 µm.

G) WB analysis quantified iNOS, CCR2, IL-1β, and IL-6 protein expression levels in aortic tissues from WT and Fap^-/-^ mice (n=6 per group; two-way ANOVA with Bonferroni multiple comparison test).

H-J) ELISA measuring expression levels of IL-1β (H), IL-6 (I), and TNF-α (J) in aortic tissues from WT and Fap^-/-^ mice after 28 days of BAPN administration (n=3 per group; unpaired two-tailed t-test).

Data are presented as mean ± standard deviation for panels B, D, and F-J.

FAP: fibroblast activation protein; BAPN: β-aminopropionitrile; TAD: thoracic aortic dissection; EVG: Elastic van Gieson; ELISA: Enzyme-linked immunosorbent assay; WT: wild-type; IL-1β: interleukin-1 beta; IL-6: interleukin-6; TNF-α: tumor necrosis factor-alpha.

**Figure S10** Fibroblast-derived Fap deletion attenuates inflammation and extracellular matrix degradation in a BAPN-induced TAD model

A) PCR analysis on genomic DNA derived from Fap^fl/fl^ mice (left), and Postn^cre/cre^ mice (right).

B) WB analysis of aortic fibroblasts from Fap^fl/fl^ and Fap^Postn^ mice with 28-day BAPN induction (n=3 per group; two-way ANOVA with Bonferroni multiple comparison test).

C, D) Representative immunofluorescence images (C) and quantitative analysis of fluorescence intensity (D) of F4/80^+^ macrophages (red) in aortic sections from Fap^fl/fl^ and Fap^Postn^ mice following 28 days of BAPN administration (n=6 fields per mouse; unpaired two-tailed t-test). Nuclei were counterstained with DAPI (blue). Scale bars, 50 µm.

E-H) Representative histological staining (Masson [E], EVG [G]) of aortic sections and quantification analysis (Masson [F], EVG [H]) between Fap^fl/fl^ and Fap^Postn^ mice after 28 days of BAPN administration (n=6 fields per mouse; unpaired 2-tailed t-test). Scale bars, 50 µm.

I) WB analysis quantified iNOS, CCR2, IL-1β, and IL-6 protein expression levels in aortic tissues from Fap^fl/fl^ and Fap^Postn^ mice after 28 days of BAPN administration (n=6 per group; two-way ANOVA with Bonferroni multiple comparison test).

J-L) ELISA measuring expression levels of IL-1β (J), IL-6 (K), and TNF-α (L) in aortic tissues from Fap^fl/fl^ and Fap^Postn^ mice after 28 days of BAPN administration (n=3 per group; unpaired two-tailed t-test).

Data are presented as mean ± standard deviation for panels B and D-L.

FAP: fibroblast activation protein; BAPN: β-aminopropionitrile; TAD: thoracic aortic dissection; EVG: Elastic van Gieson; ELISA: Enzyme-linked immunosorbent assay; WT: wild-type; IL-1β: interleukin-1 beta; IL-6: interleukin-6; TNF-α: tumor necrosis factor-alpha.

**Figure S11** Temporal changes in FAP levels and relative enzyme activity in a BAPN-induced TAD model.

A) FAP levels in aortic tissues of 3-week-old WT male mice from each group were measured by ELISA. Mice were treated with saline (control, n=12), BAPN (0.25%, wt/vol, n=12), BAPN plus Ac-Gly-BoroPro (50 μg/kg/day, n=12), or BAPN plus Ac-Gly-BoroPro (500 μg/kg/day, n=12). Measurements were taken at 7, 14, 21, and 28 days after treatment (n=3 per group each time point; two-way ANOVA with Bonferroni’s multiple comparison test).

B) Relative enzyme activity at the corresponding time points from each group (n=3 per group each time point; two-way ANOVA with Bonferroni’s multiple comparison test).

Data are presented as mean ± standard deviation for panels A and B.

FAP: fibroblast activation protein; BAPN: β-aminopropionitrile; TAD: thoracic aortic dissection; ELISA: Enzyme-linked immunosorbent assay.

**Figure S12** FAP inhibition fails to protect mice against early inflammatory infiltration and extracellular matrix degradation in a BAPN-induced TAD model.

A, B) Quantitative analysis of fluorescence intensity of FAP (A) and F4/80 (B) in aortic sections from the Saline and FAPi groups under control conditions or after 5 or 10 days of BAPN intervention (n=6 per group; two-way ANOVA with Bonferroni’s multiple comparison test).

C, D) Quantitative analysis of histological staining of Masson (C) and EVG (D) in aortic sections from the Saline and FAPi groups under control conditions or after 5 or 10 days of BAPN intervention (n=6 per group; two-way ANOVA with Bonferroni’s multiple comparison test).

Data are presented as mean ± standard deviation for panels A-D.

FAP: fibroblast activation protein; BAPN: β-aminopropionitrile; TAD: thoracic aortic dissection; EVG: Elastic van Gieson.

**Figure S13** FAP inhibitor fails to protect mice against BAPN-induced TAD formation.

A, B) Representative immunofluorescence images (A) and quantitative analysis of fluorescence intensity (B) of F4/80^+^ macrophages (red) in aortic sections from the Saline and FAPi groups following 28 days of BAPN administration (n=6 fields per mouse; unpaired two-tailed t-test). Nuclei were counterstained with DAPI (blue). Scale bars, 50 µm.

C-F) Representative histological staining (Masson [C], EVG [E]) of aortic sections and quantification analysis (Masson [D], EVG [F]) between the Saline and FAPi groups after 28 days of BAPN administration (n=6 fields per mouse; unpaired 2-tailed t-test). Scale bars, 50 µm.

G-I) ELISA measurements of IL-1β (G), IL-6 (H), and TNF-α (I) in aortic tissues from the Saline and FAPi groups after 28 days of BAPN administration (n=3 per group; unpaired two-tailed t-test).

J) WB analysis quantified iNOS, CCR2, IL-1β, and IL-6 protein expression levels in aortic tissues from the Saline and FAPi groups (n=6 per group, two-way ANOVA with Bonferroni multiple comparison test).

Data are presented as mean ± standard deviation for panels B-J.

FAP: fibroblast activation protein; BAPN: β-aminopropionitrile; TAD: thoracic aortic dissection; EVG: Elastic van Gieson; ELISA: Enzyme-linked immunosorbent assay.

**Figure S14** Differential gene expression profiles in macrophage subsets of human aortic sample from public dataset (GSE213740).​​

1. Heatmap depicting the expression levels of selected genes (listed) across distinct macrophage subtypes: MRC1^+^ Macrophages, S100A8^+^ Macrophages, IL1B^+^ Macrophages, and TOP2A^+^ Macrophages. Gene expression is normalized and scaled; red indicates high expression and blue indicates low expression relative to other cell types. ​​

B) Dotplot showcasing specific gene features within the macrophage subtypes. The size of each dot represents the percentage of cells expressing the gene (features) within a cell type (Celltype). The color intensity of the dot represents the average expression level of the gene (features) within the expressing cells in that cell type, scaled from low (light) to high (dark).

**Figure S15** Colocalization of FAP, PLAUR, and ITGB1 in human and mouse aortas.

A)​Immunofluorescence of FAP (red), PLAUR (green), and ITGB1 (pink) was assessed in aortic tissues from healthy donors and patients with acute TAD. Nuclei were counterstained with DAPI (blue). Scale bars, 100 µm.

B)​Immunofluorescence of FAP (red), PLAUR (green), and ITGB1 (pink) in aortic tissues from normal mice and the BAPN-induced TAD mouse model. Nuclei were counterstained with DAPI (blue). Scale bars, 50 µm.

FAP: fibroblast activation protein; PLAUR: plasminogen activator urokinase receptor; ITGB1: integrin beta-1; TAD: thoracic aortic dissection.

**Figure S16** FAP-PLAUR interaction promotes macrophage pro-inflammatory phenotype

A) Crystal structure of the FAP-PLAUR complex, with FAP (Chain B, green) and PLAUR (Chain A, pink).

B) Root-mean-square deviation (RMSD) analysis of individual protein chains (Chain A and B) and the interface region over a 100 ns molecular dynamics simulation; the right graph shows the RMSD distribution at the end of the simulation.

C) Molecular Mechanics/Generalized Born Surface Area method was used to calculate the binding free energy of the complex.

D) Relative fluorescence intensity of iNOS^+^ in BMDMs co-cultured with fibroblasts transfected with empty vector (EV), Flag-FAP^WT^, Flag-FAP^S624A^, and Flag-FAP^mutant^ (n=6 fields per group; one‑way ANOVA with Tukey’s post hoc test was used for multiple comparisons).

E) WB analysis quantified iNOS, CCR2, IL-1β, and IL-6 protein expression levels in BMDMs co-cultured with fibroblasts transfected with EV, Flag-FAP^WT^, Flag-FAP^S624A^, and Flag-FAP^mutant^ (n=3 per group, one-way ANOVA with Tukey multiple comparison test).

Data are presented as mean ± standard deviation for panels D and E.

FAP: fibroblast activation protein; PLAUR: plasminogen activator urokinase receptor; BMDMs: bone marrow-derived macrophages; WB: western blot.

**Figure S17** Analysis of aortic inflammation and extracellular matrix following rAAV9-mediated expression of FAP mutants in the early stage of BAPN administration

A) Experimental timeline. Male Fap^-/-^ mice were injected at 18 days with rAAV9-Postn vectors (null, FAP, FAP^S624A^, or FAP^mutant^), and BAPN administration was conducted at day 25 for 5 or 10 days before sacrifice.

B, C) Relative fluorescent intensity of Flag (B) and F4/80 (C) in different groups at indicated time points (n=6 per group; two-way ANOVA with Bonferroni's multiple comparisons test).

D, E) Quantification of the relative collagen volume fraction (D) and elastin degradation score (E) for each group at the indicated time points (n=6 per group; two-way ANOVA with Bonferroni's multiple comparisons test).

Data are presented as mean ± standard deviation for panels B-E.

rAAV9: recombinant adeno-associated virus serotype 9; FAP: fibroblast activation protein; BAPN: β-aminopropionitrile.

**Figure S18** Effects of PLAUR silencing on mRNA and protein levels.

A) Relative Plaur mRNA levels in BMDMs transfected with siRNA-scramble (si-NC), si-PLAUR#1, or si-PLAUR#2 (n=3 per group; one-way ANOVA with Tukey multiple comparison test).

B) WB and quantification analysis of PLAUR levels in BMDMs transfected with si-NC, si-PLAUR#1, or si-PLAUR#2 (n=3 per group; one-way ANOVA with Tukey multiple comparison test).

Data are presented as mean ± standard deviation for panels A and B.

BMDMs: bone marrow-derived macrophages; PLAUR: plasminogen activator urokinase receptor; WB: western blot.

**Figure S19** Administration of Vnp-16 induced no apparent organ toxicity in mice.

A) Hematoxylin and eosin-stained sections of heart, liver, spleen, lung, and kidney from saline- and Vnp-16-treated groups. Scale bars, 50 µm.

B-G) Plasma levels of alanine transaminase (ALT, B), aspartate transaminase (AST, C), creatinine (CREA, D), alkaline phosphatase (ALP, E), uric acid (UA, F), and creatine kinase (CK, G) were assessed (n=3 per group; unpaired two-tailed t-test).

Data are presented as mean ± standard deviation for panels B-G.

**Figure S20** Bar and scatter plots comparing tissue parameters in mouse models under different experimental conditions.

A, B) Quantitative analysis of histological staining (Masson [A], EVG [B]) in Fap^‑/‑^ mice treated with saline or Vnp‑16 after 28 days of BAPN administration (n=6 fields per mouse; Welch’s t‑test).

C, D) Quantitative analysis of relative fluorescence intensity (F4/80^+^iNOS^+^ [C], F4/80^+^p‑FAK^+^ [D]) in Fap^‑/‑^ mice treated with saline or Vnp‑16 after 28 days of BAPN administration (n=6 fields per mouse; Welch’s t‑test).

E, F) Quantitative analysis of histological staining (Masson [E], EVG [F]) in WT mice treated with rAAV9‑F4/80‑null or rAAV9‑F4/80‑Itgb1‑KO after 28 days of BAPN administration (n=6 fields per mouse; Welch’s t‑test).

G, H) Quantitative analysis of relative fluorescence intensity (F4/80^+^iNOS^+^ [G], F4/80^+^p‑FAK^+^ [H]) in WT mice treated with rAAV9‑F4/80‑null or rAAV9‑F4/80‑Itgb1‑KO after 28 days of BAPN administration (n=6 fields per mouse; Welch’s t‑test).

Data are presented as mean ± standard deviation for panels A-H.

FAP: fibroblast activation protein; BAPN: β-aminopropionitrile; rAAV9: recombinant adeno-associated virus serotype 9; EVG: Elastic van Gieson.

**Figure S21** Experimental workflow and validation of ITGB1 knockdown in macrophages.

A) Schematic of the study design using male WT mice. 18-day-old male WT mice were injected with rAAV9: rAAV9-F4/80-null, rAAV9-F4/80-Itgb1-KO#1, or rAAV9-F4/80-Itgb1-KO#2 to knockout Itgb1 specifically in F4/80^+^ macrophages. From 25 days of age, each group received 0.25% (wt/vol) BAPN for 28 days. After treatment, aortas were harvested for immunofluorescence staining to assess vascular lesions and macrophage infiltration. BMDMs were isolated for WB analysis.

B) WB and quantification of ITGB1 protein levels in BMDMs from each group (n=3 per group; one‑way ANOVA with Tukey’s post hoc test was used for multiple comparisons).

Data are presented as mean ± standard deviation for panel B.

ITGB1: integrin beta-1; WT: wild-type; BAPN: β-aminopropionitrile; rAAV9: recombinant adeno-associated virus serotype 9; BMDMs: bone marrow-derived macrophages; IF: immunofluorescence; WB: Western blot.


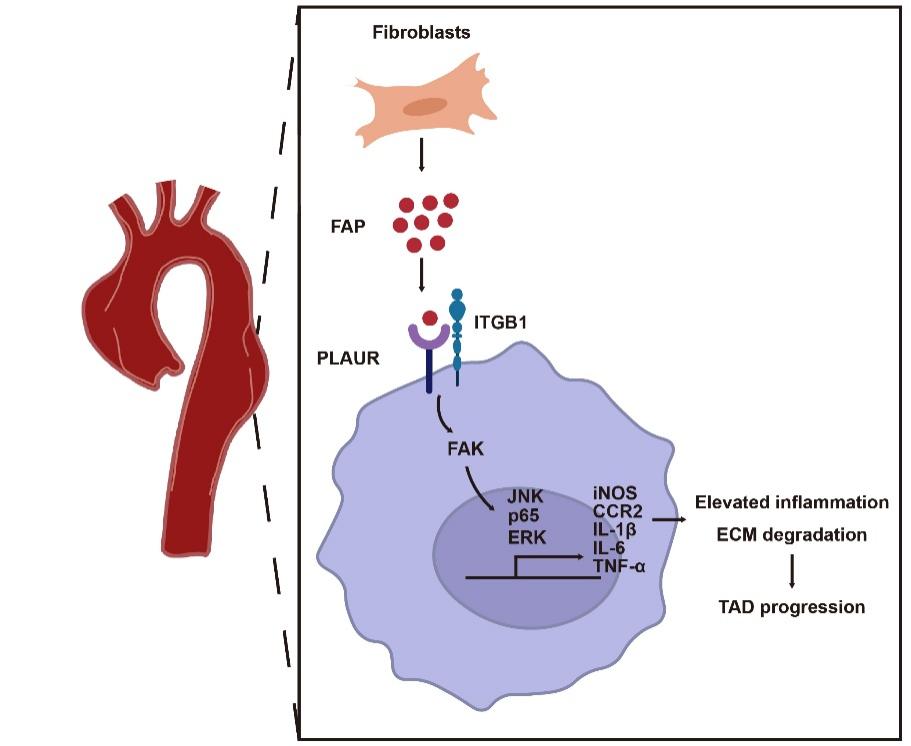


**Figure S22** FAP promotes TAD through a non-enzymatic mechanism involving fibroblast-macrophage crosstalk via the FAP/PLAUR/ITGB1/FAK axis. Inhibition of this pathway attenuates TAD in preclinical models, highlighting its potential as a therapeutic target.

FAP: fibroblast activation protein; TAD: thoracic aortic dissection; PLAUR: plasminogen activator urokinase receptor; ITGB1: integrin beta-1; JNK: c-Jun N-terminal kinase; ERK: extracellular signal-regulated kinase; FAK: focal adhesion kinase; iNOS: inducible nitric oxide synthase; CCR2: C-C chemokine receptor type 2; IL-1β: interleukin-1 beta; IL-6: interleukin-6; TNF-α: tumor necrosis factor-alpha; ECM: extracellular matrix.

**Table S1** Sequences of siRNAs.

| siRNA | Sense | Anti-sense |
| --- | --- | --- |
| si-NC | 5′-CUGACCGCAGAGGGAAUGUUU-3′ | 5′-AACAUUCCCUCUGCGGUCAG-3′ |
| si-PLAUR-1 | 5′-GCUUAGAUGUGCUGGGAAACC-3′ | 5′-UUUCCCAGCACAUCUAAGCCU-3′ |
| si-PLAUR-2 | 5′-GGAUGAGGACUACACCCGAGG-3′ | 5′-UCGGGUGUAGUCCUCAUCCUU-3′ |

**Table S2** Antibodies

| Antibody | Vendor name | Catalog number | Dilution | Application |
| --- | --- | --- | --- | --- |
| FAP-1 Polyclonal Antibody | Uptbio | PLA019661 | 1:2000 | WB |
| iNOS Rabbit Polyclonal Antibody | Beyotime | AF7281 | 1:2000 | WB |
| CCR2 Rabbit Polyclonal Antibody | Beyotime | AF6387 | 1:2000 | WB |
| IL6 Rabbit Polyclonal Antibody | Beyotime | AF7236 | 1:2000 | WB |
| IL1B Rabbit Polyclonal Antibody | Beyotime | AF7209 | 1:2000 | WB |
| Beta Actin Polyclonal antibody | Proteintech | 20536-1-AP | 1:8000 | WB |
| Plasminogen receptor Polyclonal Antibody | Uptbio | PLA019397 | 1:2000 | WB |
| Integrin β1 Polyclonal Antibody | Uptbio | PLA016482 | 1:2000;  1:1000 | WB;  IF |
| Phospho-FAK (Tyr397) Recombinant antibody | Proteintech | 83933-1-RR | 1:2000;  1:500 | WB;  IF |
| FAK Polyclonal antibody | Proteintech | 12636-1-AP | 1:2000 | WB |
| NF-κB p65 Polyclonal antibody | Proteintech | 10745-1-AP | 1:2000 | WB |
| Anti-Phospho- NF-kB p65 (S536) Rabbit pAb | Servicebio | GB113882 | 1:600 | WB |
| JNK Polyclonal antibody | Proteintech | 51151-1-AP | 1:2000 | WB |
| Phospho-JNK Recombinant antibody | Proteintech | 80435-3-RR | 1:2000 | WB |
| Anti-ERK1+ERK2 Rabbit pAb | Servicebio | GB11560 | 1:1000 | WB |
| Anti-Phospho-ERK1/2 Rabbit pAb | Servicebio | GB11004 | 1:600 | WB |
| Rabbit monoclonal to iNOS | Abcam | ab178945 | 1:250 | IF |
| Anti-CD68 antidoby | Abcam | ab53444 | 1:250 | IF |
| F4/80 Rabbit Monoclonal Antibody | Beyotime | AG4753 | 1:200 | IF |
| Vimentin Rabbit Monoclonal Antibody | Beyotime | AF1975 | 1:500 | IF |
| FAP Polyclonal antibody | Proteintech | 11779-1-AP | 1:500 | IF |
| uPAR/CD87 Polyclonal antibody | Proteintech | 10286-1-AP | 1:500 | IF |

**Table S3** Mutant FAP protein sequence

| >M509082-FAP (**E57A T58A R59A E60A S61A Y62A**)  MKTWLKTVFGVTTLAALALVVICIVLRPSRVYKPEGNTKRALTLKDILNGTFSYKTYFPNWISEQEYLHQSEDDNIVFYNI**AAAAAA**IILSNSTMKSVNATDYGLSPDRQFVYLESDYSKLWRYSYTATYYIYDLQNGEFVRGYELPRPIQYLCWSPVGSKLAYVYQNNIYLKQRPGDPPFQITYTGRENRIFNGIPDWVYEEEMLATKYALWWSPDGKFLAYVEFNDSDIPIIAYSYYGDGQYPRTINIPYPKAGAKNPVVRVFIVDTTYPHHVGPMEVPVPEMIASSDYYFSWLTWVSSERVCLQWLKRVQNVSVLSICDFREDWHAWECPKNQEHVEESRTGWAGGFFVSTPAFSQDATSYYKIFSDKDGYKHIHYIKDTVENAIQITSGKWEAIYIFRVTQDSLFYSSNEFEGYPGRRNIYRISIGNSPPSKKCVTCHLRKERCQYYTASFSYKAKYYALVCYGPGLPISTLHDGRTDQEIQVLEENKELENSLRNIQLPKVEIKKLKDGGLTFWYKMILPPQFDRSKKYPLLIQVYGGPCSQSVKSVFAVNWITYLASKEGIVIALVDGRGTAFQGDKFLHAVYRKLGVYEVEDQLTAVRKFIEMGFIDEERIAIWGWSYGGYVSSLALASGTGLFKCGIAVAPVSSWEYYASIYSERFMGLPTKDDNLEHYKNSTVMARAEYFRNVDYLLIHGTADDNVHFQNSAQIAKALVNAQVDFQAMWYSDQNHGISSGRSQNHLYTHMTHFLKQCFSLSD* |
| --- |
| >M509082-FAP (**S624A)**  MKTWLKTVFGVTTLAALALVVICIVLRPSRVYKPEGNTKRALTLKDILNGTFSYKTYFPNWISEQEYLHQSEDDNIVFYNIETRESYIILSNSTMKSVNATDYGLSPDRQFVYLESDYSKLWRYSYTATYYIYDLQNGEFVRGYELPRPIQYLCWSPVGSKLAYVYQNNIYLKQRPGDPPFQITYTGRENRIFNGIPDWVYEEEMLATKYALWWSPDGKFLAYVEFNDSDIPIIAYSYYGDGQYPRTINIPYPKAGAKNPVVRVFIVDTTYPHHVGPMEVPVPEMIASSDYYFSWLTWVSSERVCLQWLKRVQNVSVLSICDFREDWHAWECPKNQEHVEESRTGWAGGFFVSTPAFSQDATSYYKIFSDKDGYKHIHYIKDTVENAIQITSGKWEAIYIFRVTQDSLFYSSNEFEGYPGRRNIYRISIGNSPPSKKCVTCHLRKERCQYYTASFSYKAKYYALVCYGPGLPISTLHDGRTDQEIQVLEENKELENSLRNIQLPKVEIKKLKDGGLTFWYKMILPPQFDRSKKYPLLIQVYGGPCSQSVKSVFAVNWITYLASKEGIVIALVDGRGTAFQGDKFLHAVYRKLGVYEVEDQLTAVRKFIEMGFIDEERIAIWGW**A**YGGYVSSLALASGTGLFKCGIAVAPVSSWEYYASIYSERFMGLPTKDDNLEHYKNSTVMARAEYFRNVDYLLIHGTADDNVHFQNSAQIAKALVNAQVDFQAMWYSDQNHGISSGRSQNHLYTHMTHFLKQCFSLSD* |

FAP: fibroblast activation protein.

**Table S4** Primers for real-time PCR analysis in human and mice

| Gene | Forward | Reverse |
| --- | --- | --- |
| homo-FAP | 5′- ATCTATGACCTTAGCAATGGAGAATTTGT -3′ | 5′- GTTTTGATAGACATATGCTAATTTACTCCC -3′ |
| homo-β-actin | 5′-CATGTACGTTGCTATCCAGGC-3′ | 5′-CTCCTTAATGTCACGCACGAT-3′ |
| mus-Fap | 5′-GTCTCCAGCTGGGAATATTAC-3′ | 5′-TGTGCTGAGTTCTGAAAGTGC-3′ |
| mus-Plaur | 5’-GGCTTAGATGTGCTGGGAAA-3’ | 5’-CAATGAGGCTGAGTTGAGCA-3’ |
| mus-β-actin | 5′-GGCTGTATTCCCCTCCATCG-3′ | 5′-CCAGTTGGTAACAATGCCATGT-3′ |

PCR: polymerase chain reaction.
